# Dynamic control of cell state transitions during tissue morphogenesis

**DOI:** 10.1038/s44318-026-00905-x

**Published:** 2026-09-04

**Authors:** Joana E Saraiva, Sandra Edwards-Jorquera, Elias H Barriga

**Affiliations:** Biophysical Mechanisms of Morphogenesis Lab, Cluster of Excellence Physics of Life (PoL), TU Dresden, Dresden, Germany

**Keywords:** Chromatin, Transcription & Genomics, Development

## Abstract

Morphogenesis, the process by which cells, tissues, and organs acquire and maintain their form, is essential for embryogenesis, regeneration, and disease progression. During morphogenesis, cells within tissues dynamically interact with biophysical and biochemical signals from their environment by modifying their shape, gene expression, and function. These modifications often lead to transitions between cell states, and with that, to the establishment of functional programs that will in turn define embryonic stages or tissue states. Here, we discuss how the interplay of gene expression programs, chromatin modifications, and biophysical factors from the microenvironment contributes to the robust coordination of morphogenesis by allowing cells within tissues to dynamically transit across functional stages.

## Abstract (web summary)

This review summarizes the current knowledge on molecular regulation of cell and tissue state transitions in development and regeneration.

## Graphical abstract

**Figure:**
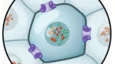

## Introduction

For embryos to develop, wounds to heal, and diseases to progress, cells within tissues need to dynamically interact with intrinsic and extrinsic biochemical and mechanical signals. The output of such interactions often leads to multiple layers of cellular responses, such as changes in cell morphology, gene expression, cell behavior, fate, and/or function. A cell’s response to a certain external environment depends on the cell’s intrinsic state, which in turn dynamically evolves due to its interaction with the environment at a given time or context. Therefore, considering the dynamic spatiotemporal evolution of the multiple factors that influence a cell´s state, the conception of a definition for “cell state” remains increasingly challenging (extensively discussed in (Rafelski and Theriot, 2024; Rukhlenko et al, 2022; Mulas et al, 2021; Miroshnikova et al, 2023; Mao and Wickström, 2024). Historically, cell states have been defined based on observable phenotypes, and definitions of cell states have evolved alongside technological advances that enabled scientists to access the cellular world at different length and time scales. For example, the first record of a compound microscope goes all the way back to 1590 and is credited to Hans and Zacharias Janssen. Later in 1665, Robert Hooke published his work “*Micrographia*” with details about an improved version of the “magnifying glasses”, or microscopes, and along with that, the first description of “minute bodies” that he named Cells (Hooke, 1665). Since then, cells became key subjects of study, with their states primarily defined by microscopic morphological features or anatomical positioning. These morphological and topological descriptions were later elaborated due to improvements made to the microscope itself by Leeuwenhoek and by the introduction of cytological or histological preparations. One of the oldest stains is Prussian blue, which was described by Max Perls in 1867 and has been used for the identification of iron-containing cells. More precise information about cell patterns, shapes, and speculation about cell functions within tissues started to be elucidated thanks to the introduction of hematoxylin and eosin stains (Hussein et al, 2015). The later inclusion of immunohistochemistry, biochemical profiling, and molecular markers would assist in more precisely relating shape, location, and function; “easing” the categorization of cell states. For example, epithelial and mesenchymal cells were defined morphologically based on their polarity, and molecularly, by the protein content of their junctions (i.e., cadherins). Additionally, the emergence and rapid evolution of molecular sequencing approaches have led to the idea that a specific and stable cell state is the output of transcriptional gene-regulatory networks. These networks are proposed to coordinate the activity of transcription factors (TFs), signaling pathways, and chromatin regulation, to define a specific gene expression profile (Rafelski and Theriot, 2024).

The idea of a stable cell state solely defined by its transcriptional profile has become increasingly challenging to sustain, as recent developments in multi-omics and live imaging approaches reveal the presence of transient or intermediate cell states (MacLean et al, 2018). This, in addition to the natural variations within cell populations due to spatial heterogeneities among cell collectives, as well as considering all possible factors at play (e.g., biomechanical (Villeneuve et al, 2024; Mongera et al, 2018; Marchant et al, 2022; Balasubramaniam et al, 2024; Calvo et al, 2013) or bioelectrical (Ferreira et al, 2025b; Cohen et al, 2014)), suggests that no two cells would be in an exactly identical state. Adding to this, chromatin accessibility and epigenetic regulation can stabilize (or destabilize) intermediate programs, store “memory” of past signals, and constrain future responses (Bell et al, 2024), discussed in detail in “Chromatin and the control of gene expression and cell states”.

Accordingly, what constitutes a cell state and a transition between states is constantly being reassessed by researchers. To date, one of the most accepted definitions proposes that “a cell state is defined by the cellular ability to perform specific functions”, and that the transition between states entails a “detectable change in function” (Miroshnikova et al, 2023). This idea emphasizes that cell states reflect a combination of particular molecular, functional, and contextual variables. In this review, we use “cell state” as the integrated molecular, functional, and contextual condition of a cell, and we refer to “transitions” as sustained changes in function.

Regardless of how we define them, cells transition between several states during morphogenesis to ensure the appropriate spatiotemporal coordination of events during development and regeneration, and such transitions are also relevant for the maintenance of tissue homeostasis in adults (Pera and Rossant, 2021; Brandão et al, 2022; Singh et al, 2022; Stower et al, 2023). Furthermore, when dysregulated, they may lead to pathologies (Acloque et al, 2009; Oswald et al, 2017; Park et al, 2015; Pastushenko and Blanpain, 2019; Patel and Yanai, 2024), hence the clinical potential of studying cell state transitions. Thus, this review presents an integrated view of the mechanisms controlling dynamic cell state transitions during embryonic development and tissue regeneration.

### Cell state transitions during embryonic development and regeneration

The study of morphogenesis can be traced back to the ancient Greek philosophers, such as Aristotle, who described the rival theories of organism generation and development: Epigenesis, which he favored, states that the well-organized whole (i.e., the embryo) develops gradually from an undifferentiated mass in a sequence of steps. Pangenesis, on the other hand, states that every part of the adult body contributes some specific material to the seed. The concept of pangenesis evolved later to the idea of Preformation, which affirmed that germ cells contain a preformed miniature version of an adult, that simply becomes larger during development (reviewed in (Van Speybroeck et al, 2002)). As embryologists and anatomists leveraged the progress of light microscopy tools to observe, compare, and manipulate developing embryos, epigenesis became the favored explanation among embryologists again. Although first proposed many centuries ago, this concept reflects the wide range of morphological and functional changes that occur during the transition from a single undifferentiated cell to the complexity of a fully developed organism. Nowadays, it is evident that for these changes to occur, cells undergo a series of important state transitions.

The very first cell state transition during ontogeny occurs at fertilization, when gamete fusion generates a totipotent zygote (Schindler-Johnson and Petridou, 2024). Subsequently, a species-specific number of cell divisions, along with changes in cell adhesion and gene expression, establishes the pre-gastrulation embryo (often referred to as a blastula or blastocyst, depending on the organism) (Le Verge-Serandour and Turlier, 2022). In many externally developing embryos, this transition coincides with the classic mid-blastula transition (MBT), initially described by changes in cell proliferation dynamics, asynchrony of cell divisions, and the transition from non-migratory to migratory cell types. Later, the inclusion of molecular approaches into the study of embryo development uncovered that these changes coincide with large-scale zygotic genome activation (ZGA) involving the transition from a development program controlled by maternally deposited mRNAs and proteins to one controlled by the zygotically transcribed RNAs, referred to as the maternal-to-zygotic transition (MZT) (reviewed in (Tadros and Lipshitz, 2009; Vastenhouw et al, 2019)). In mammals, analogous molecular transitions occur with different timing and without a canonical MBT framework.

**Figure 1:**
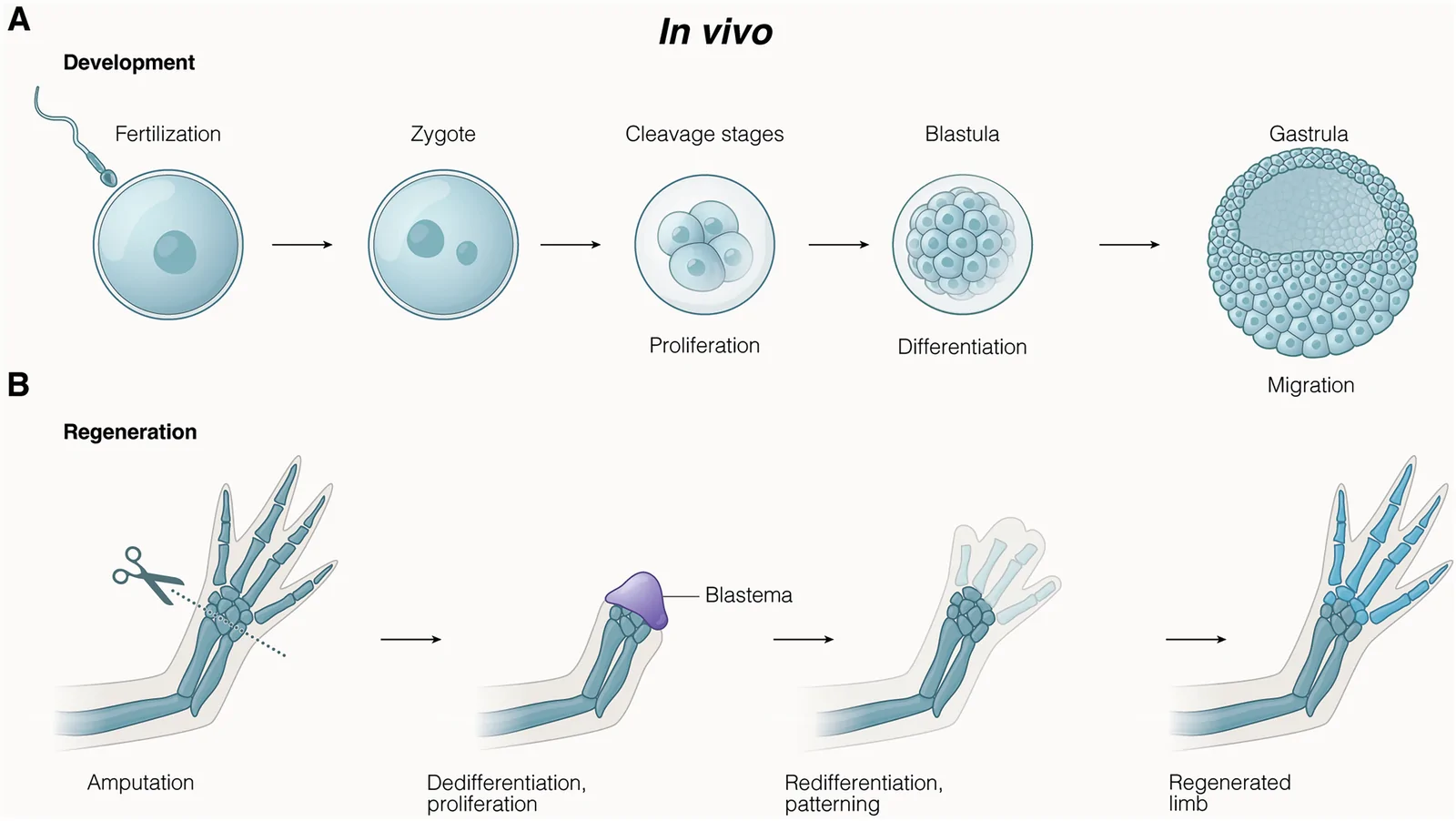
Cell state transitions during embryonic development and regeneration in vivo. (**A**) During embryonic development, fertilization generates a zygote that undergoes a series of cell divisions during cleavage stages, differentiation during blastula formation, and subsequent gastrulation, when cells migrate into new positions and contribute to germ-layer formation. (**B**) General progression from an amputated to a fully regenerated limb in salamanders. Following amputation, a blastema forms via cell dedifferentiation/recruitment of quiescent cells, followed by proliferation. The blastema will give rise to most missing structures via redifferentiation into the same or different cell types (e.g., fibroblast into cartilage), and tissue patterning, which is accompanied by tissue growth.

Following this, a new wave of changes in cell states appears with the onset of gastrulation. Gastrulation is an essential embryonic stage, as the germ layers of the embryo—endoderm, mesoderm, and ectoderm—and the main body plan are established (Steventon et al, 2021). During gastrulation, different layers of the embryo will transit into specialized cell states to support a combination of collective cellular behaviors, such as collective cell migration and epithelial-to-mesenchymal transition (EMT), priming the transition towards the onset of neurulation (Fig. 1A). These examples represent a small subset of transitions that occur in a developing embryo which, in many contexts, are accompanied by progressive cell differentiation, thus reducing the available spectrum of cell fates. However, it is not clear how these events—among many others—rely on the ability of cells to adapt and change, often acquiring new morphologies and molecular scenarios that enable them to perform new functions.

Unlike embryonic development, during regeneration, the starting point is a fully patterned tissue, and cells may adopt different paths depending on their context and species. Some quiescent cells regain proliferative potential in response to damage, while some mature cells dedifferentiate into multipotent progenitor cells or directly transdifferentiate into other cell types (Tanaka and Reddien, 2011). Overlapping with the burst in cell proliferation, multiple developmental programs become reactivated. With this, missing tissues are rebuilt and patterned into fully functional structures, whereby stem cells become restricted in their lineage and positional identity (Kragl et al, 2009; Otsuki et al, 2025). Although regeneration has been proposed to recapitulate developmental programs (Roensch et al, 2013; Gerber et al, 2018), it has also been shown that distinct mechanisms may take place (Masselink et al, 2024; Aztekin, 2024; Johnson et al, 2022).

For regeneration to occur, different mechanisms take place depending on the degree of damage, the structure being affected, and the species-specific regeneration potential. During epimorphic regeneration, for example, all tissues that are lost upon amputation are regrown in a patterned fashion by de novo morphogenesis. This process is mediated by a blastema or regenerative bud, which are specialized structures from where most missing tissues will emerge. Blastemas arise from the cells close to the wound site that undergo cell dedifferentiation and/or recruitment of tissue-resident stem cells via proliferation and/or cell migration and become morphogenetic organizing units (Londono et al, 2018) (Fig. 1B). This type of regeneration has been classically studied in non-amniote vertebrates (amphibians and fish) (Nakatani et al, 2007; Stocum, 2013), but is also present with some limitations in mammals (Dolan et al, 2022). Some invertebrates possess even more striking regenerative abilities, such as flatworms and cnidarians, as they are able to regenerate whole organisms from small tissue fragments (Reddy et al, 2019; Aboobaker, 2011). Nevertheless, regardless of the species and tissue-specific regeneration potential, cells undergo dynamic state transitions, as reflected by changes in cell proliferation, identity, and migratory properties during this process (Tanaka and Reddien, 2011; Friedl and Gilmour, 2009; Marconi et al, 2021). More broadly, regeneration also highlights a fundamental trade-off between specialization and plasticity. Indeed, differentiation and maturation often improve tissue performance, but can restrict proliferative potential and regeneration. A clear example is the mammalian heart, where cardiomyocytes largely lose the ability to divide and thus heal an injured heart, to ensure greater contractile performance upon maturation (Porrello et al, 2011). Across vertebrates, the proliferative state of cardiomyocytes is thus directly linked to their ability to regenerate cardiac tissue.

The fact that during embryonic development, a single-celled zygote gradually transitions across the aforementioned stages (Ingram and Keane, 1980), may lead to the assumption that cell state transitions are inherently unidirectional, and culminate in stable cell states. However, classic embryological experiments already distinguished between specification and commitment. For example, in ectopic and/or heterochronic grafting assays, tissues that are merely specified can be redirected to alternative fates by a new environment, whereas committed cells largely maintain their original identity (Solini et al, 2017). In addition, the striking ability of some organisms to naturally reprogram their identity and reactivate developmental programs to regenerate (Tanaka and Reddien, 2011; Ke et al, 2025; Muneoka and Bryant, 1982; Jopling et al, 2011) further supports the idea that, when the appropriate conditions are met, at least some cell state transitions may be reversible. Beyond these natural phenomena, the discovery that differentiated somatic cells can be experimentally reset to a pluripotent-like state by defined TFs provided direct mechanistic evidence that cell states are, at least in part, reversible (Takahashi and Yamanaka, 2006).

### Reversible cell state transitions

**Figure 2:**
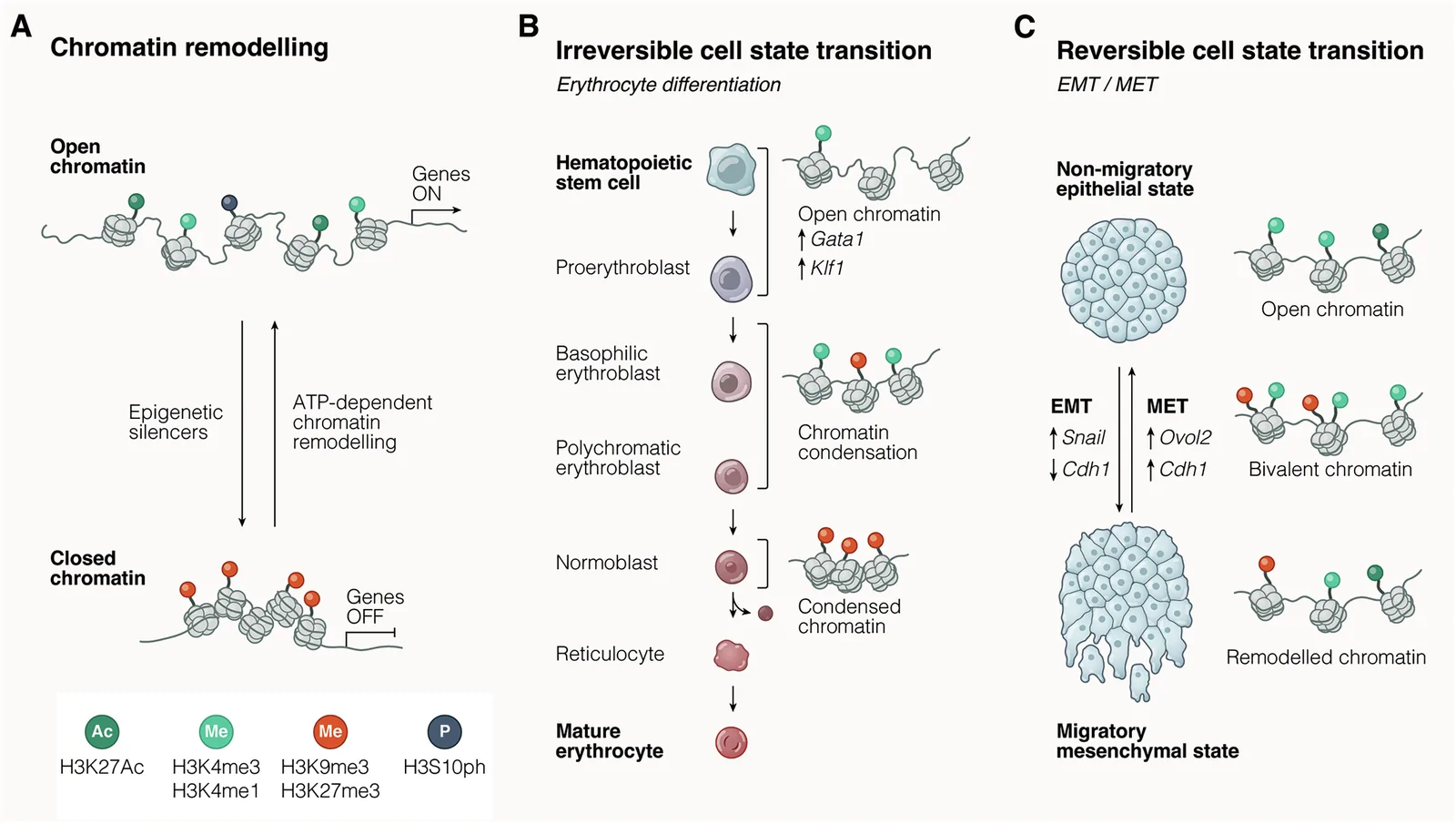
Chromatin remodeling during irreversible and reversible cell state transitions. Chromatin states are regulated by combinations of histone modifications, DNA methylation, chromatin-remodeling complexes, and transcription factor activity. (**A**) Schematic representation of chromatin remodeling. Open and transcriptionally permissive regions are commonly associated with marks such as H3K27ac and H3K4me1,3, whereas repressed chromatin is associated with marks such as H3K9me3, H3K27me2,3, and DNA methylation. (**B**) Irreversible cell state transition during mammalian erythroid differentiation. Erythroid transcription factors such as Gata1 and Klf1 activate lineage-specific gene expression while chromatin progressively condenses as cells mature toward reticulocytes and enucleated erythrocytes. (**C**) Reversible and context-dependent cell state transitions during epithelial-to-mesenchymal transition (EMT) and mesenchymal-to-epithelial transition (MET). During EMT, epithelial programs, such as *cdh1*, are repressed while mesenchymal regulators such as *snail* can be induced. During MET, epithelial regulators such as *ovol2* and *cdh1* are reactivated. In contrast to irreversible transitions, these chromatin and transcriptional states remain dynamically reversible.

Cell state transitions were originally conceived as linear and irreversible processes, particularly in cases involving permanent genome alterations, such as V(D)J recombination during lymphocyte maturation (Christie et al, 2022; Kraft and Rubin, 2016), or the loss of the nucleus during terminal erythrocyte differentiation in mammals (Newton et al, 2024) (Fig. 2A,B), as well as the sequential gene expression and cell movements during cardiac development (Glickman and Yelon, 2002). However, partly based on a better understanding of the dynamic regulation of chromatin accessibility, it is now clear that some cell types can fluently transit across states depending on the context (Fig. 2A,C), thereby retaining the potential to correct, remodel, re-pattern, or regenerate when needed.

Although sometimes used interchangeably, a cell in transition does not necessarily correspond to an intermediate state. An intermediate cell state is a relatively stable state that lies between two better-defined stable states and can eventually represent a bona fide cellular program, for example, lineage-restricted multipotent progenitors between pluripotent stem cells and fully differentiated cells, or hybrid epithelial and mesenchymal states during EMT that exhibit mixed features (Sha et al, 2019) (Fig. 2A–C). In contrast, a transitional cell state typically refers to an unstable state between two other stable ones, moving through a transitory process from one cell type to another. In single-cell datasets, intermediate and transitional states may be transcriptionally hard to discriminate, sharing overlapping programs. Still, intermediate states more often appear as a distinct, well-defined cluster, whereas transitional states more commonly manifest as a continuum between clusters (Trapnell et al, 2014).

When linking state to function, cell competence is often a particularly relevant form of intermediate state (Gilbert 2000). For example, a pre-migratory cell is able to migrate in response to permissive cues. A migratory cell exhibits a distinct behavior, but this is enabled by prior priming through an underlying migration-competent state (Lencer et al, 2021). Other examples of cell competence include muscle satellite cells, which are primed for myogenesis because Myf5 mRNA is already present, yet quiescence is maintained by blocking translation via microRNA-31 sequestration in mRNP granules until activation cues are met (Crist et al, 2012). A related, broad mechanism is promoter-proximal RNA polymerase II stalling, enabling rapid gene activation of repressed or poised genes during development upon appropriate cues (Zeitlinger et al, 2007). In many systems, competence or priming is also supported by chromatin features such as poised regulatory elements and bivalent domains, which carry both activating and repressive marks. Finally, multilineage transcriptional priming, first described in hematopoiesis in 1997 as the co-expression of lineage-affiliated programs prior to commitment (Hu et al, 1997), has since then been observed in many other systems (Illingworth et al, 2016; Li et al, 2020; Delorme et al, 2009). Beyond primed cell states, fate transitions are often governed by regulatory cross-antagonisms: mutually repressive gene-regulatory circuits that resolve multipotent or transcriptionally mixed states into discrete outcomes, such as during segmentation “gap” genes in Drosophila (Kraut and Levine, 1991); or dorsal–ventral neural tube patterning in vertebrates (Briscoe et al, 1999; Balaskas et al, 2012). Such repressive circuits can be further stabilized via epigenetic mechanisms, including Polycomb-associated silencing, helping lock particular fates and providing heritable stability across divisions. However, such stabilization is neither necessarily stagnant nor permanent, and can be context-dependent, allowing chromatin remodeling and fate plasticity. Indeed, the fluid nature of cell state has been described as a set of “clouds,” “continuum,” “unfolding,” and “dynamics,” emphasizing how diverse states flow over time, across space, and shift throughout a single cell’s development (Mak, 2017).

Even though scRNA-seq is a powerful tool to examine cell heterogeneity previously obscured by bulk measurements, transcriptomic profiles alone are imperfect proxies for state, as they rather represent snapshot measurements. As a result, inferred state transitions can be inflated or obscured by biasing factors, including clustering and trajectory-inference parameters, batch effects, dropout, and over-smoothing, which can create or erase apparent trajectories between clusters (Kiselev et al, 2019). Importantly, not all molecular changes have functional consequences: many may reflect stochastic gene expression or local context without manifesting a phenotype (Swain et al, 2002). Thus, it remains challenging to determine whether the differences we detect truly indicate biological fluidity. A way to provide functional relevance is to complement sequencing approaches with time-resolved sampling, RNA velocity, or kinetic inference (Bergen et al, 2020), lineage tracing (Wagner and Klein, 2020), genetic perturbations, and protein or epigenetic readouts to validate proposed transitions (Stoeckius et al, 2017). Therefore, cell states are characterized not only by their stability (persistence in the absence of continued input), but also by their plasticity (the propensity to switch to an alternative state) and reversibility (the ability to return to a previous state).

Reversible transitions typically involve changes in gene expression that do not necessarily require permanent modifications to the chromatin, thus allowing cells to transiently adapt to developmental and environmental cues. On the other hand, irreversible transitions reflect a permanent change in cellular identity or function, which can be enforced by genome rearrangements, such as V(D)J recombination, or by more permanent alterations in chromatin state or gene expression via feedback controls or epigenetic modifications (Blanco et al, 2021). Epigenetic memory includes a sustained response to stimuli through maintained epigenetic states. These states can persist through cell division and maintain a specific epigenetic state involving chromatin-modifying complexes (Simon and Tamkun, 2002). However, chromatin regulation does not, on its own, determine whether transitions are reversible; rather, it can constrain or enable reversibility by shaping how readily gene-regulatory programs can be reconfigured. This includes mechanisms such as histone modifications, chromatin remodeling, such as Polycomb-associated repression/heterochromatinization (Steffen and Ringrose, 2014), DNA methylation that is often heritable and stable (Kim and Costello, 2017), and non-coding RNAs (ncRNAs), which modulate gene expression and thereby influence cell identity and function (see “Chromatin and the control of gene expression and cell states”). However, other changes may be dynamically regulated. For example, during early mammalian development, pluripotent cells of the preimplantation epiblast are able to self-renew and to differentiate into the three embryonic germ layers, which is typically unidirectional in vivo (Lawson and Pedersen, 1992; Tam and Loebel, 2007). In vitro, embryonic stem cells (ESCs) that are derived from the preimplantation epiblast/inner cell mass provide a complementary model system to study the underlying mechanisms. Molecularly, pluripotent stem cells are characterized by the expression of the TFs *Oct4*, *Nanog*, *Sox2*, and *Klf2/4* (Nichols and Smith, 2009). However, the ectopic expression of such factors (excepting *Nanog*) in differentiated somatic cells, along with the oncogene *c-Myc*, can genetically reprogram them into an embryo-like state, which is why these cells have been termed induced pluripotent stem cells (iPSCs) (Takahashi and Yamanaka, 2006; Takahashi et al, 2007). The transition from differentiated somatic cells into iPSCs typically involves chromatin remodeling to a more accessible configuration that favors the expression of pluripotency genes (Mattout et al, 2011). However, such process is considered reversible, as under the right conditions, iPSCs can differentiate again into various other cell types (Takahashi and Yamanaka, 2006; Takahashi et al, 2007). Reversible changes in cell identity are also observed during epimorphic regeneration, where some cells interconvert between fates via reprogramming processes like dedifferentiation, re-differentiation, and transdifferentiation (reviewed in (Jopling et al, 2011)).

In addition to changes in cell fate and identity, cells may also transition in their migratory behavior, which is exemplified by epithelial-to-mesenchymal and mesenchymal-to-epithelial transitions (EMT and MET, respectively) (Haerinck et al, 2023). The concept of EMT has been subject to redefinition as new molecular and physical observations become available, much like the definition of cell state transitions (reviewed in (Yang et al, 2020)). In a simplified view, EMT involves the progressive loss of epithelial cell polarity and cell-cell adhesion, while gaining migratory properties, often related to a mesenchymal phenotype. On the other hand, MET involves the acquisition of apico-basal polarity and the formation of stable epithelial-like adherens junctions in the formerly mesenchymal cells. Both EMT and MET play pivotal roles during different stages of development and regeneration, as well as in the initiation and progression of pathologies such as cancer metastasis (Marconi et al, 2021; Chuai et al, 2012; Pei et al, 2019; Acevedo et al, 2007; Mani et al, 2008). For example, during early development, sequential MET and EMT processes take place, where MET is associated with an initially reversible phase, in which cells initiate their exit from pluripotency to form embryonic epithelia (Hamidi et al, 2020). Also, sequential EMT and MET transitions are required for somatic cell reprogramming into iPSCs (Liu et al, 2013). Accordingly, the balance between EMT and MET has proven to be key for “healthy” cells to transition into neoplastic states (Forte et al, 2017). Furthermore, the evidence of embryonic (Dong et al, 2018) and neoplastic (Pastushenko et al, 2018) cells displaying attributes of both epithelial and mesenchymal phenotypes supports the existence of transient intermediate EMT and MET cell states. EMT also plays key roles in vertebrate development, such as allowing neural crest cells to transit from non-migratory to migratory cell states (Leathers and Rogers, 2022). Interestingly, this transition between migratory and non-migratory cell states can be reversibly instructed by biophysical cues emerging from the microenvironment (Marchant et al, 2022; Barriga et al, 2018; Saraiva et al, 2025), and recent data have shown that minor changes in mechanical cues can alter chromatin state and reversibly prime neural crest cells for migration (Saraiva et al, 2025). Similar biophysical control is also observed during tumorigenesis and *Drosophila* embryogenesis (Farge, 2003). In pathology, cancer metastasis often involves irreversible EMT, during which cells adopt a more plastic, stem-cell-like phenotype, associated with increased migratory and invasive properties (Acevedo et al, 2007; Mani et al, 2008).

The initiation or termination of cell movement may also be explained from a physical perspective, as changes in mechanical and geometric parameters can trigger a shift between solid-like (jammed) and fluid-like (unjammed) states, in some contexts without requiring immediate transcriptional alterations (Mongera et al, 2018; Oswald et al, 2017; Pajic-Lijakovic and Milivojevic, 2023). Therefore, akin to the abovementioned transitions, the conversion between jammed and unjammed states can be reversible or irreversible, depending on the context (Blauth et al, 2021). However, unjamming/jamming transitions may differ from EMT/MET in certain contexts, such as the case of changes in the collective migration of epithelial cells (Mitchel et al, 2020). A clear example of an irreversible jamming transition is observed during vertebrate body axis elongation, where posterior tissues shift from fluid-like properties in the mesodermal progenitor zone to a solid-like behavior in the presomitic mesoderm (Mongera et al, 2018). Unjamming transitions have been strongly associated with tumor progression (Oswald et al, 2017; Gottheil et al, 2023; Blauth et al, 2021). Studying the multiple angles underlying the reversibility of intermediate cell states and their regulation may hold the key to generate an integrated view of the mechanisms that allow robust transitions between embryonic stages, the acquisition of regenerative cell states, and the transition from healthy to neoplastic conditions.

## Molecular regulation of cell/tissue state transitions

Cellular behavior is determined by a combination of extrinsic and intrinsic factors. Here, we discuss the contributions of chromatin accessibility, gene expression, and their regulation by environmental factors in the induction of intermediate cell state changes. This prepares the arena for discussions on how these mechanisms are integrated to synchronize morphogenetic transitions in multicellular systems (“Biophysical synchronization of cell state transitions in multicellular contexts”).

### Gene expression and cell states

Gene expression has been widely used to define cell states and identities, since it is subjected to highly controlled changes in response to intra- and extracellular signals. Gene products, depending on their nature, can act as instructive or responsive factors when contributing towards a given cell state. Regulatory genes are characterized by being activated in response to specific developmental signals, often possessing stable expression patterns, and guiding cells toward certain fates or identities (Pollex et al, 2024). Effector genes, on the other hand, can be rapidly modulated by different cues, and their expression is often transient, thereby allowing flexibility and adaptation to changes in the cell microenvironment. Both types of genes are essential for proper tissue function and contribute to the complex dynamics of cell identity.

Genes playing instructive roles in early mammalian development are exemplified by core pluripotency TFs. In the preimplantation epiblast, *Oct4, Sox2*, and *Nanog* help establish and maintain pluripotency. In vitro, mouse ESCs recapitulate key aspects of this program and express these factors, together with naive pluripotency regulators such as *Klf2/4* (Nichols and Smith, 2009). Forced expression of defined factors, classically *Oct4, Sox2, Klf4*, and *c-Myc*, in differentiated somatic cells can reprogram them into iPSCs, restoring pluripotency-like potential (Takahashi and Yamanaka, 2006; Takahashi et al, 2007). During epimorphic regeneration, some cell types regain multipotency, such as blastema cells (reviewed in (Tamura et al, 2010)), which derive from the dedifferentiation of mesodermal tissues directly underneath the wound epithelium, although their specific source depends on the species and regenerating body segment (Knopf et al, 2011; Muneoka et al, 1986; Sandoval-Guzmán et al, 2014; Wang et al, 2012). For example, during limb regeneration in the axolotl, blastema cells regain an embryonic gene expression program comparable to limb bud cells, being characterized by the expression of Homeobox genes (*Prrx1, Msx, Hoxd10, Hoxd11*), critical for patterning and positional identity; T box genes (*Tbx4, Tbx5*), important for limb identity and outgrowth; morphogens (*Shh, Fgf8, Fgf10*), providing positional information; while also expressing a blastema specific gene (*Kazald2*, originally termed *Kazald1*), required for regeneration to progress (Bryant et al, 2017; Khan et al, 2002; Torok et al, 1999; Stewart et al, 2013). Similarly, the froglet limb blastema cells express Homeobox genes (*msx1* and *prx1*), T-box genes (*tbx5*), and morphogens (*shh, fgf8, fgf10*) (Endo et al, 1997, 2000; Suzuki et al, 2005), with equivalent functions as described above. The zebrafish caudal fin blastema cells express genes coding for Homeobox genes (*msxe, dlx5a*), TFs (*junbl*), chromatin remodeling enzymes (*smarca4*); and chaperones (*hspa9*), which have proven to be necessary for larval fin regeneration to proceed (Kawakami et al, 2004; Yoshinari et al, 2009; Ishida et al, 2010). The combined expression of these genes (among others) in the appropriate spatial and temporal scales promotes cell state changes like dedifferentiation, proliferation, redifferentiation, and cell migration. Consequently, the coordination of their expression in time and space is required for the transition between cell states that will secure correct morphogenesis during regeneration. Several of these genetic cascades of transcriptional regulators and effector pathways are first used by embryos to develop, and their degree of conservation or overlap is now the focus of intense research (reviewed in (Sinigaglia et al, 2022; Levine and Davidson, 2005)).

### Chromatin and the control of gene expression and cell states

The different roles that specific gene products have on cell state transitions depend, in part, on the mechanisms that govern the induction and maintenance of their expression, and such mechanisms are strongly linked to the regulation of chromatin accessibility. Hence, studying how gene expression is regulated in response to chromatin conformation changes is essential to understand how cell states and their transitions are controlled. Several mechanisms can influence the way that the transcriptional machinery accesses regulatory elements to modulate gene expression (Valencia and Kadoch, 2019; Webster and Phillips, 2025; Chen et al, 2024). For example, changes in chromatin accessibility and three-dimensional architecture can lead to large-scale alterations in gene expression through the formation of topologically associated domains (TADs) and loops, that facilitate the interaction between regulatory domains and their target genes (Boltsis et al, 2021). Furthermore, chromatin is organized into distinct compartments that correlate with either active or repressive chromatin modifications, between which entire TADs can shift, thus influencing gene expression changes, and consequently, cell state transitions (Dixon et al, 2015). For instance, Polycomb repressive complexes (PRCs), which are responsible for many of the repressive histone modifications, can lead to gene silencing and chromatin compaction, and can thus greatly influence cell states (Guo and Wang, 2022). Such repression is crucial, for example, for the differentiation of pluripotent stem cells (Leeb et al, 2010). Pioneer factors, a subclass of TFs, on the other hand, can bind to silent chromatin regions and induce their opening, thus driving cell fate transitions (Barral and Zaret, 2024; Cirillo et al, 2002) such as the MZT (Chen et al, 2023). Accordingly, dysregulation of these pioneer TFs may lead cells to transit into altered states, such as cancer, due to aberrant cell reprogramming (Barral and Zaret, 2024).

Chromatin post-translational modifications (PTMs) are associated with permissive or repressive chromatin states and can influence transcription by modulating chromatin accessibility, nucleosome dynamics, and the recruitment of chromatin-associated factors. For example, lysine acetylation neutralizes positive charge on histone tails, which can weaken interactions with DNA and is frequently associated with transcriptionally permissive chromatin. In addition, histone modifications can serve as binding platforms for “reader” proteins and chromatin-modifying or remodeling complexes (Patel, 2016), for instance, bromodomain-containing factors that recognize acetylated lysines (Marmorstein and Zhou, 2014). Consistently, certain histone modifications are frequently enriched at active or repressed regulatory elements and influence cell states in both development and disease (Janssen and Lorincz, 2022). Because these marks – which occur mostly in the amino-terminal region of histones– often act in combination, their interplay has been discussed as “the histone code” (reviewed in (Jenuwein and Allis, 2001)). The histone code does not only refer to methylation and acetylation, but also to sumoylation (Ryu and Hochstrasser, 2021), ubiquitylation (Vaughan et al, 2020), citrullination (Zhu et al, 2021), serotonylation (Farrelly et al, 2019), dopaminylation (Lepack et al, 2020), and glycation (Mir et al, 2021), among others (Zheng et al, 2025; Huang et al, 2018; Zhao et al, 2019).

An additional epigenetic mechanism that influences gene expression is DNA methylation, which is a heritable yet reversible conversion of cytosine to 5-methylcytosine by DNA methyltransferase (DNMT) enzymes. The altered cytosine residues are usually immediately adjacent to a guanine nucleotide, called CpG or CG sites, which results in two methylated cytosine residues sitting diagonally to each other on opposing DNA strands. It was initially thought that this modification only played a role in repressing gene expression, but it has become clear now that they are highly dynamic, and that proper DNA methylation patterns are essential for cell differentiation and embryonic development (Suzuki and Bird, 2008). Consequently, defective methylation patterns have been associated with several diseases and birth defects (Cao et al, 2021; Nishiyama and Nakanishi, 2021; Ulschmid et al, 2024). Furthermore, histone modifications may help targeting DNA methylation during embryonic development, and vice versa (Rose and Klose, 2014; Fujimori et al, 2026), which has shown to be relevant for cell fate determination (Marks et al, 2012; Habibi et al, 2013).

A clear example of chromatin modifications associated with changes in cell state occurs during early embryonic development, where the initial pluripotent state is gradually lost, while lineage commitment and cell fate-associated genes begin to be expressed (Fisher and Fisher, 2011; Sun et al, 2021). In the mouse embryo, pluripotent epiblast cells initially exhibit a relatively permissive chromatin and low DNA methylation landscape that becomes progressively restricted as development proceeds (Smith et al, 2012; Wu et al, 2016), which is associated with increased expression of DNMTs (Auclair et al, 2014). This increase in global DNA methylation levels is crucial for cell fate commitment and differentiation, as pluripotency-associated genes and cell type-specific markers are silenced to avoid the expression of “non-appropriate” lineage genes (Kalkan et al, 2017; Xie et al, 2013). Inversely, DNA methylation marks are largely erased during epigenetic reprogramming of iPSCs (Lister et al, 2009; He et al, 2017) and primordial germ cells (Messerschmidt et al, 2014). These changes in DNA methylation patterns are also accompanied by histone modifications (Li et al, 2012; Shanak and Helms, 2020; Christophorou et al, 2014).

Upon lineage specification, the new cell identity should be stably maintained during cell division. Hence, a global gain of DNA methylation as well as loss of methylation at specific *loci* occurs during differentiation in a cell-type-dependent manner (Suelves et al, 2016; Calvanese et al, 2012; Kim et al, 2011; Nazor et al, 2012). Although after a cell divides, its daughter cells do not always inherit the same epigenetic information, as is the case of asymmetric cell divisions, which are key to ensure appropriate development and organism homeostasis (reviewed in (Chen et al, 2018; Zion and Chen, 2021)). Understanding how epigenetic marks are inherited has opened a wide field of research which is nowadays the focus of both experimental and theoretical groups (Mukherjee et al, 2026; Zion et al, 2020).

Whereas embryonic development mainly involves progressive cell-fate specification, tissue regeneration may additionally require differentiated cells to regain plasticity and adopt alternative fates (reviewed in (Jopling et al, 2011)), suggesting that some epigenetic signatures require resetting. In line with this, it has been proposed that the extent to which an organism can regenerate is not dictated by the presence or absence of the necessary genes, but rather by whether such genes are appropriately expressed following injury, which may correlate with the plasticity of its epigenome (Katsuyama and Paro, 2011). For example, frogs—which possess limited regenerative abilities—do not reactivate Shh expression in limb blastemas (Yakushiji et al, 2007), whereas the more regeneration-competent axolotls do (Torok et al, 1999), with these gene expression differences being associated with methylation levels at the Shh *locus*. Along with epigenome plasticity, some epigenetic constraints must remain to ensure that the regenerated structure is appropriately patterned, such as the case of positional identity during limb regeneration (Otsuki et al, 2025; Nacu et al, 2013), which is epigenetically encoded along the proximodistal axis in connective tissue cells via repressive histone marks in homeoprotein gene loci (Kawaguchi et al, 2024).

Identifying damage-responsive enhancers that regulate regenerative gene expression in multiple species and tissues may provide mechanistic insights into the epigenome plasticity of regenerating animals. Several such enhancers have been described (Kawaguchi et al, 2024; Kang et al, 2016; Suzuki et al, 2019; Wang et al, 2020b), some of which are reused from developmental programs, while others are unique to regeneration (reviewed in (Harris, 2022)), and it has been proposed that regeneration-specific enhancers have lost their activity in non-regenerating species (Suzuki and Ochi, 2020). Epigenetic silencing of enhancers may also contribute to the age-dependent loss of regenerative abilities in different tissue types and species, as shown in *Drosophila* imaginal wing discs (Harris et al, 2016) and mouse auditory hair cells (Tao et al, 2021), where regenerative abilities are lost at specific developmental stages.

The relevance of the mechanisms underlying the regulation of gene expression in defining cell states is clear, as well as their fundamental contribution to the transition across intermediate cell states, and even the reversibility of such transitions. Yet, how do all these mechanisms—discussed here—“know” where and when to activate gene expression? In other words, what is the “epi” in the control of our genetic programs? Studying this is essential if one aims to understand what synchronizes cell state transitions in tissues that are exposed to varying environmental conditions.

### Microenvironment and the regulation of chromatin state and gene expression

**Figure 3:**
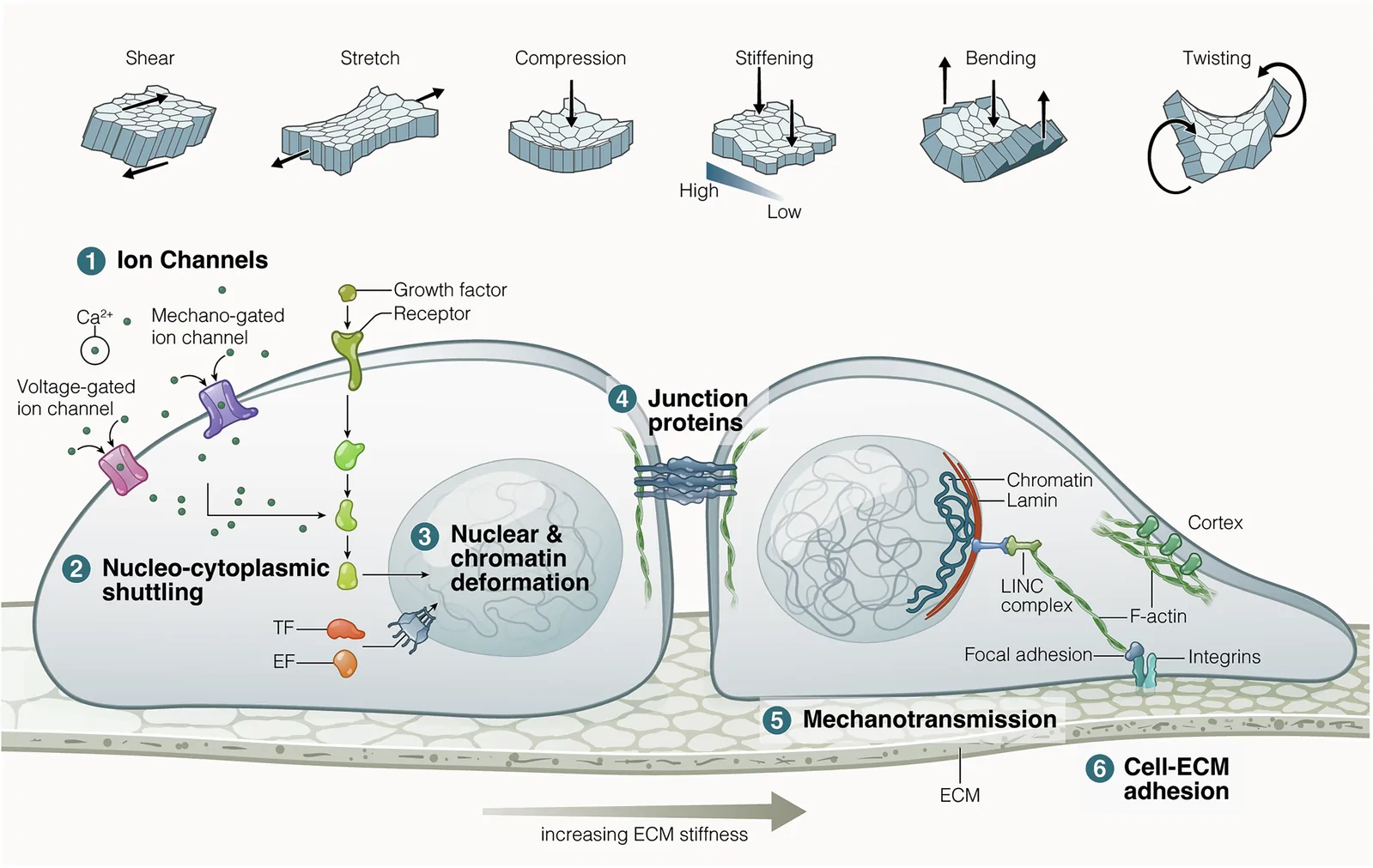
Routes for transmission of biophysical inputs to chromatin and gene expression. Multiple cellular structures can sense and transmit biophysical cues within cells, between neighboring cells, and between cells and the surrounding extracellular matrix (ECM). Tissue-scale inputs such as shear, stretch, compression, stiffness gradients, bending, and twisting can be translated into cellular responses through several routes. (1) At the plasma membrane, mechanosensitive and voltage-gated ion channels convert external stimuli into ionic fluxes, such as Ca²⁺ entry, while growth factor receptors can further cooperate to induce signaling. (2) Downstream signaling can regulate nucleo-cytoplasmic shuttling of epigenetic factors (EFs), transcription factors (TFs), and other signaling mediators. (3) In parallel, biophysical inputs can deform the nucleus and chromatin, potentially altering chromatin organization, accessibility, and transcriptional output. (4) Mechanical and biochemical inputs can also be transmitted between neighboring cells through junctional complexes, including adherens and tight junctions. (5) Forces can also be conveyed from the cell cortex and cytoskeleton to the nucleus through F-actin, the LINC (linker of nucleoskeleton and cytoskeleton) complex, and the nuclear lamina, coupling cytoskeletal force transmission to nuclear architecture. (6) At the cell–ECM interface, integrins and focal adhesions sense and respond to extracellular matrix properties such as stiffness, ligand presentation, and applied forces, and convert them into intracellular mechanotransduction. Together, these routes enable cells to integrate diverse mechanical information with biochemical signaling to regulate chromatin state and gene expression.

Gene expression is regulated by genetic and epigenetic factors that influence cell behavior and play key roles in intermediate cell state transitions. In our days, epigenetics have been referred to as the heritable changes in gene expression that do not involve altering the DNA sequence, associated with a combination of chemical modifications to histones and DNA molecules performed by remodeling enzymes (Ngo and Hein, 2021), as well as to the activity of regulatory ncRNAs (Wei et al, 2017). Still, cells remodel their chromatin and adjust their gene expression in response to environmental factors such as nutrient availability, temperature shifts, hypoxia, pathogens, drugs, osmotic stress, and mechanical forces (Dupont and Wickström, 2022; Pudelko and Cabianca, 2024). Many of these stimuli are relevant when defining cell states in multiple biological contexts. For example, biochemical signals, including morphogens, cytokines, hormones, and growth factors, as well as exogenously administered drugs or vitamins, bind to receptors and activate specific signaling pathways. These pathways often lead to chromatin remodeling and changes in gene expression that control cellular processes which are essential for tissue morphogenesis like survival, size, proliferation, differentiation, and migration, among others (Li et al, 2021; Liu et al, 2022; Maden, 1982; Mercader et al, 2000; Glaviano et al, 2023; Göke et al, 2013; Mir et al, 2023; Valle-Mendiola et al, 2023; Yang et al, 2012, 2024; Takebayashi-Suzuki and Suzuki, 2020; Elsherbiny and Dobreva, 2021) (Fig. 3). In the case of morphogens, it has been proposed that the previous exposure to these ligands provides competence for cells to respond to subsequent cues, easing the progression across states. An example of this type of memory is the case of Wnt signaling, which is a necessary step for cells to respond to Activin during gastrulation (Yoney et al, 2018). It is tempting to speculate that such memory or “primed state” may be mediated by epigenetic mechanisms.

Indeed, during *Xenopus* gastrulation, the Wnt/β-catenin signaling pathway is thought to prime gene expression in the embryonic organizer—the structure from where the dorsal axis develops. Mechanistically, the authors found that Wnt signaling recruits the histone methyltransferase Prmt2 to the regulatory elements of a series of target genes, where it allows for methylation of arginine residues (H3R8me). This methylation is thought to mark regulatory elements of these genes for their later recognition by the transcriptional complex that will mediate their expression (Blythe et al, 2010). A similar scenario has been found in *Drosophila* (Hannon et al, 2017). These examples illustrate how chromatin regulation can be influenced by external factors to render the chromatin a ‘poised’ or marked state, which will facilitate subsequent events of gene expression governing cell state transitions.

Several vitamins are also able to influence epigenetic landscapes, thus impacting gene expression and consequently, cell phenotypes (reviewed in (Leesang et al, 2024)). B vitamins, such as B2 (riboflavin), B6, B9 (folate), and B12 (cobalamin), are considered to be pro-methylating micronutrients. This is mostly due to their role in one-carbon metabolism, which generates S-adenosylmethionine, the sole methyl donor used by all methyltransferases to methylate proteins and nucleic acids, thus influencing chromatin modifications and genomic stability (reviewed in (Fustin, 2022)). For this reason, an imbalanced one-carbon metabolism has been associated with developmental defects and disease progression (Lyon et al, 2020). For example, an inadequate intake of folate during early stages of pregnancy may lead to neural tube defects in the offspring (Gurugubelli and Ballambattu, 2024), and folic acid supplementation during later developmental stages has been associated with changes in DNA methylation and changes in the expression of neural target genes (Ondičová et al, 2022). Whether these effects are due to a direct regulation remains to be addressed. Also, vitamin C has been strongly linked to the activity of ten-eleven translocation (Tet) proteins, a family of enzymes that catalyze the oxidation of 5-methylcytosine, thus promoting DNA demethylation (Brabson et al, 2021; Hore et al, 2016). Vitamin A (all-trans retinol) and its active metabolites, such as retinoic acid (RA), collectively called retinoids, are involved in regulating cell state transitions by controlling several aspects of cell proliferation, differentiation, and survival (Gudas and Wagner, 2011; Kukreja et al, 2020; Monaghan and Maden, 2012; Nguyen et al, 2017; Persaud et al, 2016; Moreno-Oñate et al, 2024). This is achieved primarily through ligand-dependent transcriptional control by retinoic acid receptors (RAR/RXR), which bind RA response elements (RAREs) and recruit coactivator/corepressor and chromatin-modifying/remodeling complexes, thereby coupling RA-responsive gene regulation to local chromatin remodeling (reviewed in (Urvalek et al, 2014)). For example, RA binds to the RA receptor (RAR) family of TFs, which interact with RA response elements (RAREs) within target gene promoters and enhancers, which are essential to define cell states during development (Balmer and Blomhoff, 2002). Several changes in gene expression related to RA have proven to be context-dependent. During regeneration, for example, RA can reprogram blastema cells to a proximal identity but fails to do so in fully patterned limbs or after redifferentiation has commenced (McCusker et al, 2014; Niazi et al, 1985).

Aside from biochemical cues, biophysical properties of cells and their surrounding environment can also regulate cell state transitions. For example, acting across tissues, cells, organelles, and even individual molecules, mechanical forces exert fundamental effects on cell behavior through the regulation of gene expression. These forces are highly tissue-specific and include compression, shear stress, tensile stress, and hydrostatic pressure (reviewed in (Dupont and Wickström, 2022)). Their generation, transmission, and persistence depend not only on the applied stimulus itself but also on the mechanical properties of tissues and cells, such as elasticity, viscosity, and stiffness, as well as on the physical properties of the extracellular matrix (ECM) (Chaudhuri et al, 2020). The impact of such mechanical forces on cell state transitions can be fine-tuned, as they depend on their magnitude, direction, frequency, and duration (Amar et al, 2021). Furthermore, mechanical forces not only modulate cell behavior and gene expression but can also directly or indirectly regulate epigenetic modifications that, in some contexts, persist after stimulus removal and generate forms of context-dependent “mechanical memory” (Kalukula et al, 2025; Scott et al, 2023a; Yang et al, 2014; Scott et al, 2023b).

The influence of other biophysical stimuli, such as low oxygen levels (hypoxia), bioelectrical signals (Koga et al, 2019), heat stress (Zhang et al, 2023; Fischl et al, 2020), and osmotic shock (Amat et al, 2019) or pollutants, has also been shown to influence chromatin dynamics, gene expression, and consequently, cell function. In summary, environmental signals of different natures establish bidirectional feedback between different cellular structures that sense and respond to them while shaping cell morphology, chromatin conformation, and transcription. However, to fully comprehend their role in the regulation of cell state transitions, it is necessary to consider not only these signals individually, but also how they interact within complex native environments and contribute to emergent collective cell behaviors.

## Biophysical synchronization of cell state transitions in multicellular contexts

In a living system, all mechanisms discussed until this point, and many others yet to be discovered, coexist and rarely act on isolated cells, eventually assisting cells within tissues to transit across states and to respond to future stimuli (Yoney et al, 2018; Scott et al, 2023b). Recent evidence indicates that this interplay between regulatory mechanisms not only ensures that cells change states, but also coordinates that cell transitions occur at the right time and place. For instance, in morphogen-driven patterning, tissue-scale gradients of diffusible ligands bias fate decisions in a position-dependent manner while maintaining multipotent progenitor states (Kicheva and Briscoe, 2023).

**Figure 4:**
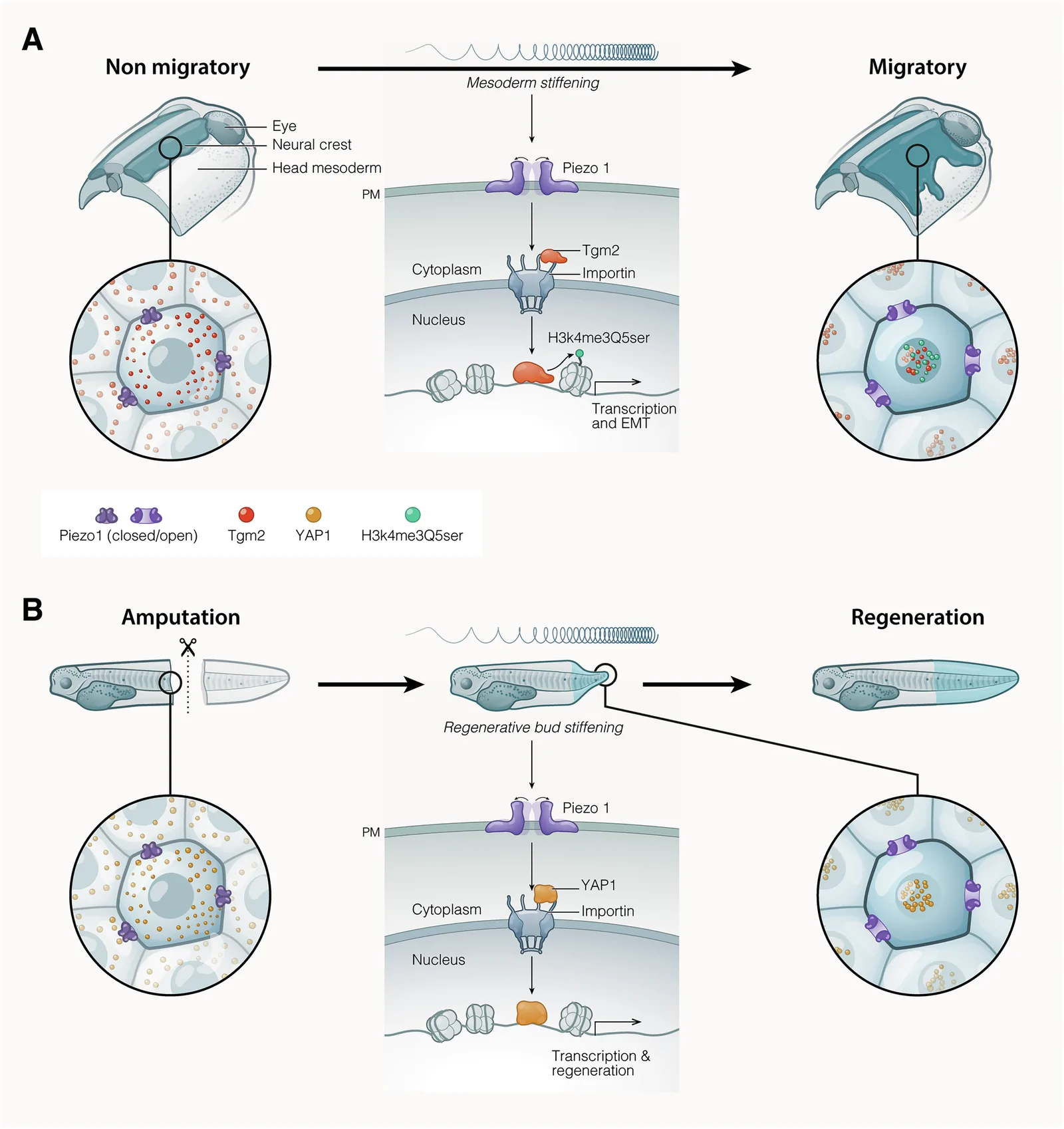
Tissue interplay during development and regeneration. (**A**) Mechanistic overview of the onset of *Xenopus* cranial neural crest migration in vivo. Pre-migratory cells sense and respond to stiffening of the underlying head mesoderm. Mesoderm stiffening activates the mechanosensitive ion channel Piezo1, promoting intracellular calcium signaling and favoring the active conformation of Tgm2 and its interaction with importins. This enables the nuclear translocation of Tgm2, where it catalyzes histone serotonylation (H3K4me3Q5ser) to fine-tune chromatin state and promote the transcriptional program required for a migratory switch. (**B**) Schematic of *Xenopus* tail regeneration highlighting a recently identified mechanical state transition during the progression from wound healing to regenerative bud formation. At this stage, prospective bud tissue undergoes strain stiffening, which activates a Piezo1–Yap1 mechanosensitive axis. Tissue stiffening promotes Piezo1-dependent nuclear localization of Yap1, leading to activation of a regenerative program that allows wounded epithelial tissues to transit into a competent-bud state to drive epimorphic regeneration.

To translate mechanical inputs into molecular responses that drive morphogenetic transitions, cells rely on specialized structures that sense local forces through dedicated proteins or protein complexes, a process termed mechanosensing (Vogel and Sheetz, 2006; Yap et al, 2018). These cues are then converted into intracellular biochemical and structural responses through mechanotransduction, including pathways such as RhoA/ROCK, MAPK, calcium signaling (Martino et al, 2018), and mechanoresponsive transcriptional regulators such as YAP/TAZ, MRTF–SRF, β-catenin, and NFκB (reviewed in (Dupont and Wickström, 2022)). Through interconnected systems such as adhesion complexes, the cytoskeleton, mechanosensitive ion channels, and the nucleus, mechanical inputs can thus be transmitted to influence chromatin regulation and gene expression (Figs. 3 and  4A). Early evidence that external factors can induce molecular changes to modulate gene expression, and with that, the coordination of morphogenetic processes, was shown during *Drosophila* development (Farge, 2003). Here, the author analyzed the impact of mechanical stress application on the expression of the EMT TF *twist* (Farge, 2003). The expression of *twist* is typically localized to ventral territories due to the action of the morphogen Dorsal. The author then showed that the application of 10% uniaxial deformation to the embryos induces ectopic *twist* expression downstream of the mechanically triggered nuclear translocation of Armadillo, a β-catenin homolog. Later, this concept was extended by showing that endogenous tissue deformations during germ-band extension upregulate *twist* expression in the stomodeal primordium (Desprat et al, 2008).

Another example illustrating the coupling of tissue-level interactions with the emergence of mechanical forces is the mechanically triggered transition between non-migratory and migratory states of Xenopus cranial neural crest cells. The collective migration of these cells is triggered by stiffening of the underlying mesoderm, which is caused by increased cell density as a result of convergent extension during gastrulation (Barriga et al, 2018). Mesoderm stiffening is thought to be sensed by neural crest cells via Piezo1 and integrin-mediated cell-to-substrate adhesions. These molecules lead to neural crest cytoskeletal modifications, that in turn soften these cells, allowing them to initiate their collective migration (Marchant et al, 2022). Recent work has further shown that mechanical interplay across tissues can control chromatin state and morphogenetic transitions, as stiffening of the mesoderm primes the overlying neural crest cells for migration in vivo by inducing histone serotonylation (Fig. 4A). Specifically, this mechanical input promotes the nuclear translocation of transglutaminase 2 (Tgm2), an enzyme that catalyzes histone serotonylation, thereby modifying the chromatin landscape to favor gene transcription and drive the transition from non-migratory to migratory cell states (Saraiva *et al*, 2025). More broadly, these findings highlight how tissue-level mechanics can be conveyed to the nucleus to prime cell state transitions. Another route of force transmission involves the direct propagation of actomyosin-generated tension to the nucleus through the linker of nucleoskeleton and cytoskeleton (LINC) complex at the nuclear envelope (Amar et al, 2021; Rothballer et al, 2013) (reviewed in (Dupont and Wickström, 2022)). Such rapid mechanotransmission can modulate nuclear transport dynamics (Van Steensel and Belmont, 2017), the nuclear accumulation of mechanosensitive transcriptional regulators such as YAP/TAZ, NFkB, SRF, AP-1 (Dupont and Wickström, 2022; Andreu et al, 2022), as well as chromatin organization (Andreu et al, 2022; Mammoto et al, 2012; Mendez and Janmey, 2012). In addition, the nucleus is not only mechanosensitive but can also deform in response to applied stress to protect the genome (Guilluy et al, 2014; Nava et al, 2020). This direct force transduction pathway is exemplified by human mesenchymal stem cells (MSCs), which decondense their chromatin when cultured on stiff substrates and redistribute heterochromatin away from the nuclear periphery (Killaars et al, 2018). Such chromatin remodeling events can guide cell state transitions, as they bias stiffness-driven MSC differentiation potential to the osteogenic lineage (Killaars et al, 2020).

Another process in which coordinated mechanical forces between tissues guide cell state transitions is the development of mammalian hair follicles. This process is initiated by the formation of an epithelial invagination termed a placode, which coincides with the emergence of a designated hair follicle stem cell population. Seminal work showed that mechanical stress initiates the formation of the structure and the acquisition of its molecular identity, owing to a mechanically-driven nuclear translocation of β-catenin (Shyer et al, 2017). Later studies showed that the contractile forces from surrounding fibroblasts help shape the epithelial structures of the hair follicle placode, while the generated localized mechanical stresses further enhance compartmentalization of *Sox9* expression, which promotes stem cell positioning (Villeneuve et al, 2024). Such a mechanical effect on stem cell differentiation is consistent with previous work linking tension to cell fate progression through changes in chromatin organization in vitro (Miroshnikova et al, 2018). Consequently, it is feasible that mechanically induced chromatin conformation changes also underlie cell fate transitions during mammalian hair follicle development in vivo.

A mechano-molecular coupling has been shown during the elongation of the vertebrate body axis as well. Authors showed a jamming-to-unjamming transition in which N-cadherin allows for tissues in the presomitic mesoderm to flow and rearrange, while keeping their mechanical tissue integrity (Mongera et al, 2018). Later work explored how genetic factors affect the physical states of different posterior tissues, and with that, zebrafish body axis elongation (Stooke-Vaughan et al, 2025). The authors found that different mutants that target tissues required for body axis elongation—such as *noto*, *tbx16*, and *vangl*2—affect the fluidity of these tissues. This work complements the previous examples, as it highlights the contribution of genetic factors to the physical states of cells and tissues.

As discussed above, during regeneration, the interaction between several tissue types coordinates cell state changes required for proper cell dedifferentiation, proliferation, and patterning of the missing structures (reviewed in (Bassat and Tanaka, 2021)). For instance, urodele limb amputation induces rapid re-epithelialization, during which cells from the surrounding epidermis spread across the exposed stump and establish a wound epithelium within ~12 h (Hay and Fischman, 1961). Unlike intact bilayered skin, this wound epithelium is mainly composed of keratinocytes and lacks a basement membrane, facilitating interactions between the epithelium and the underlying stump tissues (Iten and Bryant, 1973; Repesh and Oberpriller, 1978). The wound epithelium subsequently thickens and develops into the apical epithelial cap, a specialized signaling center that promotes blastema growth and pattern formation during regeneration (Boilly and Albert, 1990; Tsai et al, 2020). It has been proposed that the apical epithelial cap is formed due to nerve-induced dedifferentiation of keratinocytes (Satoh et al, 2008) into a limb bud-like state through epigenetic modifications (Aguilar and Gardiner, 2015). The apical epithelial cap interacts with axons to create the appropriate biochemical niche for blastema development (Kumar et al, 2007, 2011), and nerve-derived signals are required for blastema cells to proliferate (Brockes, 1984; Mescher and Tassava, 1975). However, nerves alone can only sustain blastema proliferation for a while, as an additional position-related input is necessary to complete regeneration. This is nicely exemplified by the “accessory limb model”: If a peripheral nerve is cut and rerouted to a skin wound on a lateral site of the limb, it can evoke the formation of an ectopic blastema, which will only lead to the formation of an accessory limb if a piece of complementary limb tissue is grafted to the wound site. However, in the absence of anterior/posterior tissue interaction, proliferation and further regeneration are not sustained, and the ectopic blastema will degenerate with time (Endo et al, 2004; Lheureux, 1977; Satoh et al, 2007). Hence, an artificial juxtaposition of anterior and posterior limb tissues combined with innervation is necessary and sufficient to induce complete regeneration of an ectopic patterned limb. The requirement for such anterior–posterior tissue interactions is based on the complementary cross-inductive signals provided by both tissues, which are required for limb outgrowth and patterning—Fgf8 from anterior and Shh from posterior tissues (Nacu et al, 2016). However, it has also been proposed that components of the ECM also play an important role in the induction of pattern formation (Phan et al, 2015; Vieira et al, 2021). The accessory limb model is a simplified model system that allows us to uncover what tissues or molecular signaling components are sufficient for the growth of new limbs during regeneration (Endo et al, 2004). Therefore, the dynamic intercellular interactions enabling blastema formation, proliferation, and differentiation in this model are likely regulated by the same gene expression changes and epigenetic modifications as limb regeneration, albeit on a different scale (Min and Whited, 2022).

Akin to embryonic development, mechanical forces emerging in regenerating tissues, as well as externally applied forces, may also contribute greatly to the regulation of stem cell self-renewal and differentiation potential, along with cell migration and survival during regeneration (reviewed in (Chiou et al, 2018; Vining and Mooney, 2017)). Such mechanical stimuli can lead to epigenetic modifications that induce reversion of mature cells towards developmental-like states (Ransom et al, 2018). For example, during mammalian jaw regeneration, mechanical signals derived from bone separation are transduced via the focal adhesion kinase (FAK) signaling in bone-residing stem cells, activating neural crest-associated gene-regulatory networks required for new bone to form (Ransom et al, 2018). The transcriptional coactivator Yap was also found to be required for appendage regeneration, by inducing cell proliferation in the axolotl (Bay et al, 2023), zebrafish (Mateus et al, 2015), and *Xenopus* (Hayashi et al, 2014b). Accordingly, it has been shown that TEAD4—the TF to which Yap binds in order to induce gene expression—plays an equivalent function in *Xenopus* tail regeneration (Hayashi et al, 2014a). Although the role of Yap in regeneration is indisputable, whether its activity is directly linked to mechanical stress has remained unclear. Recent work now suggests that Piezo1–Yap signaling translates tissue mechanics into a transcriptional program that confers regenerative competence and is required to coordinate progression from wound healing to regenerative bud formation (Ferreira et al, 2025a) (Fig. 4B). Altogether, these findings highlight how adhesion complexes, mechanosensitive ion channels, and force-responsive transcriptional regulators constitute interconnected routes through which physical signals can regulate cell state transitions.

Among these mechanosensory systems, G protein-coupled receptors (GPCRs), ion channels, and receptor tyrosine kinases provide a rapid route to sense membrane stress and convert it into downstream biochemical signaling events (Xiao et al, 2023). GPCRs can transmit various external and internal cues across the cell membrane, including mechanical stress (Lin et al, 2022; Zou et al, 2004; Schnitzler et al, 2008). For example, the Angiotensin II type 1 receptor is mechanosensitive in a ligand-independent manner, evoking an intracellular signaling cascade via G proteins that eventually leads to hypertrophy in cardiomyocytes (Zou et al, 2004). Aside from GPCRs, mechanosensitive ion channels such as Piezo1 and 2, first discovered in 2010 (Coste et al, 2010) are arguably the best recognized mechanosensors involved in several signaling pathways in a wide plethora of contexts and tissue types (Segel et al, 2019; Malko et al, 2023; Wang et al, 2020a; Xiao, 2024; Xiao et al, 2024; Zhou et al, 2020). An interesting example of these channels playing a role in cell state transitions demonstrated that Piezo1/2 are required as force sensors during bone development by facilitating the expression of target genes that induce osteoblast differentiation (Zhou et al, 2020). Although GPCRs have been linked to epigenetic gene regulation (Armando et al, 2022; Shu et al, 2015), it is thus far unclear whether mechanically activated GPCRs can also lead to chromatin modifications. Conversely, mechanosensitive ion channels have been linked to epigenetic regulation. For example, Piezo1 activation in intestinal goblet cells was reported to reduce SUV39H1-associated H3K9me3 and H3K9me3 occupancy at the *mucin2* promoter under mechanical stimulation (Xu et al, 2023). A Piezo1–RhoA–ROCK axis has also been linked to tumor progression through epigenetic mechanical memory (Boyle et al, 2026), with compression-induced, ROCK-dependent histone modifications and sustained gene activation. TRPM7, an ion channel-kinase, has likewise been shown to generate nuclear kinase fragments that associate with chromatin remodeling machinery, including Polycomb group proteins, influencing histone modifications and gene expression programs (Krapivinsky et al, 2014). Together, these findings support the idea that mechanosensitive channels do not merely relay physical information to the cytoskeleton, but can also contribute more directly to chromatin-based regulation of cell state.

The cellular microenvironment and interplay between cell types also play a major role in cell state changes in pathological conditions, as is the case of tumor progression, where tumor growth can exert mechanical pressure on the non-tumorous surrounding tissue, triggering β- catenin signaling, thus influencing gene expression and cell proliferation. Hence, not only genetic and biochemical factors, but also mechanical forces can change the cellular microenvironment and contribute to cancer progression (Fernández-Sánchez et al, 2015). Furthermore, cancer-associated fibroblasts (CAFs)—a heterogeneous cell population that differs phenotypically and functionally from normal fibroblasts, which is associated with important genetic and epigenetic alterations (Du and Che, 2017; Kehrberg et al, 2023; Schmidt et al, 2024)—have the ability to modify the mechanics and architecture of the tumor microenvironment (Barbazan et al, 2023; Talayero and Vicente-Manzanares, 2023). Therefore, by modifying ECM stiffness, and regulating immune cell recruitment and inflammatory responses, CAFs may exert either tumor-suppressive or tumor-supportive functions, which influence invasiveness and responsiveness to therapies, thus making them potential therapeutic targets (Talayero and Vicente-Manzanares, 2023; Forsthuber et al, 2024; Sahai et al, 2020). Consistently, other disorders such as atherosclerosis, arthritis, osteoporosis, cardiomyopathies, and several developmental diseases are associated not only with altered mechanics but also with abnormal cellular responses to mechanical force (Di et al, 2023; Dupont and Wickström, 2022).

All things considered, the interaction between different cell types is significantly influenced by environmental cues and plays a crucial role in regulating morphogenetic transitions. These cues, such as signaling molecules and physical stimuli from the surrounding environment, can trigger chromatin reorganization, which is essential for facilitating access to gene-regulatory regions and thus impacting gene expression. Ultimately, understanding how the dynamic interplay between intercellular communication and environmental factors influences chromatin remodeling and gene expression may provide critical insights into mechanisms regulating physiological and pathological changes in cell states during development, regeneration, and disease.

## Perspective

We have reviewed examples of different biological contexts where cells interpret and respond to multiple signals, leading to coordinated changes in chromatin accessibility, gene expression, and cell behavior. Recent advances are beginning to address these issues, suggesting that gene expression enabling state transitions is not solely driven by enzymes modifying chromatin, but also by the concerted action of biochemical and biophysical stimuli that affect the subcellular localization and activity of epigenetic factors (Dupont and Wickström, 2022; Pudelko and Cabianca, 2024). Furthermore, we provided examples demonstrating that in a complex environment, the synergistic action of multiple levels of control synchronizes robust morphogenetic transitions to drive what we interpret as self-organized morphogenesis. Given the wide biological relevance of understanding how different regulatory mechanisms define robust morphogenetic programs, the topic is currently under intense research and is one of the current focuses of discussion in the field (Mulas et al, 2021; Miroshnikova et al, 2023; Mao and Wickström, 2024; Ke et al, 2025; Kukreja et al, 2024; Mulas, 2024).

An interesting question that emerges from this review is whether reversible transitions actually progress in the opposite way as they initially did, or follow a different trajectory. Furthermore, whether cells truly reach their initial state after such transitions requires further elucidation. Another emerging question is how cells undergo a broad range of transitions or intermediate states before fully altering their identity or fate commitment (Antolović et al, 2019; Mojtahedi et al, 2016). For example, canonical chromatin marks such as methylation and acetylation were shown to modulate gene expression to determine major changes in cell fate (Beacon et al, 2021; Benayoun et al, 2014). Newly identified chromatin marks—such as histone serotonylation and dopaminylation— are known to regulate small subsets of target genes (Zhu et al, 2021; Farrelly et al, 2019; Lepack et al, 2020; Mir et al, 2021; Zheng et al, 2025; Huang et al, 2018). Hence, it is tempting to hypothesize that these marks may fine-tune chromatin landscapes, thus enabling gene expression programs required for cells to transit across intermediate states before fully committing to a functional cell state. The role of these newly identified chromatin modifications is only beginning to be addressed in vitro. Thus, larger efforts are needed to fully exploit their contribution to the acquisition of intermediate and potentially reversible cell states. Even larger efforts are required to dissect whether and how small chromatin marks respond to changes in the microenvironments and/or whether they are part of an intrinsic program. Regardless of the herculean nature of the tasks ahead, we expect to have highlighted the urgent need to bring fields together and study how fluctuating cellular microenvironments can exploit mechanisms of gene expression to achieve reversible and intermediate states to ultimately drive robust morphogenesis. These efforts have the potential to pave the way towards a more comprehensive view of cell state transitions, leading to the development of better diagnostic tools, innovative therapies, and a deeper understanding of life itself.

## Supplementary information

Peer Review File
