# Supplementary material for: Dynamic control of cell state transitions during tissue morphogenesis
Source: EMBO J. 2026 Sep 4;45(21):7326–46. doi: 10.1038/s44318-026-00905-x (PMC13728214; doi:10.1038/s44318-026-00905-x)
Supplement: Supplementary file 1 [file 44318_2026_905_MOESM1_ESM.pdf]

THE EMBO JOURNAL

Dynamic control of cell state transitions during tissue morphogenesis

Joana Saraiva, Sandra Edwards-Jorquera, and Elias Barriga

Corresponding author: Elias Barriga (elias.barriga@tu-dresden.de)

Review Timeline:

Submission Date:	20th Oct 25
Editorial Decision:	16th Dec 25
Revision Received:	2nd Jun 26
Editorial Decision:	2nd Aug 26
Revision Received:	14th Aug 26
Accepted:	19th Aug 26

Editor: Ieva Gailite

Transaction Report:

(Note: With the exception of the correction of typographical or spelling errors that could be a source of ambiguity, letters and reports are not edited. Depending on transfer agreements, referee reports obtained elsewhere may or may not be included in this compilation. Referee reports are anonymous unless the Referee chooses to sign their reports.)

Dear Elias,

Thank you for submitting your review article to The EMBO Journal. I apologise for the protracted assessment process due to delays in referee report submission. We have now received reports from two experts, which are included below for your information.

As you will see, while both referees per se appreciate the topic of the article, they also find that its substantial extension and deepening of the discussion via inclusion of a broader spectrum of relevant articles, especially in a more complex developmental context, would be needed. From the editorial side, this also applies to the comments of reviewer #2 on the mechanotransduction part, instead of a full removal of this section. Furthermore, they find that more attention should be paid to the use of precise terminology. Finally, they indicate that the informative value of the figures needs to be significantly expanded.

If you were willing to commit to such a substantive revision along the lines indicated by the reviewers, I would be willing to consider a revised version of the article. In this case, I might send it back to the reviewers to obtain their further input.

From the editorial side, I have also included in the attachment further details on figure preparation for the final version. I would be happy to discuss the revision further.

I look forward to your revision and to working together with you on the improvement of your review.

With kind regards,

Ieva

Ieva Gailite, PhD
Senior Scientific Editor
The EMBO Journal
Meyerhofstrasse 1
D-69117 Heidelberg
Tel: +4962218891309
i.gailite@embojournal.org

We realize that it is difficult to revise to a specific deadline. In the interest of protecting the conceptual advance provided by the work, we recommend a revision within 3 months (16th Mar 2026). Please discuss the revision progress ahead of this time with the editor if you require more time to complete the revisions.

Referee #1:

SUMMARY
In this review entitled "Dynamic control of cell state transitions during embryo development and regeneration", Saraiva et al. consider the concept of cell state, especially its multimodal nature from chromatin dynamics to cellular behaviors, and the extent to which states are reversible as a function of cell-intrinsic properties and cell-extrinsic influences, whether biochemical or mechanical.
After introducing the concept of cell state being multimodal through a historical lens, connecting the concept to (cellular) function, the authors first survey classic transitions, including the maternal-to-zygotic and epithelium-to-mesenchyme transitions during development, and contrast with regeneration-specific transitions that follow a different, sometimes opposite direction from differentiated to undifferentiated states.
The authors then pivot to discussing the degrees to which specific transitions are reversible, which is an intuitive premise to regenerative capabilities, providing a rich suite of examples covering various modalities of more-or-less plastic transitions, from chromatin states to cellular behaviors.
The authors then proceed to visiting the molecular underpinnings of cell state transitions, starting with gene expression followed by chromatin states and the impact of the cell microenvironment on state transitions, providing an extensive review of the relevant literature.
Finally, the authors place the cell-centric concept of state into a multiscale perspective by discussing how cell state transitions contribute to tissue-scale development and morphogenesis.

The authors provide 4 Figures to illustrate their points: Figure 1 is a rather general and simple illustration of embryonic development and regeneration, Figure 2 illustrates various transitions in cell states and multicellular contexts, Figure 3 illustrates example of biochemical and mechanical signal transduction mechanisms impacting chromatin states and gene expression, Figure 4 illustrates the tissue context in which external/mechanical properties influence state transitions.

KEY MERITS AND GENERAL EVALUATION
This review addresses an active area of research, providing a very thorough, creative and inspiring perspective, covering a broad range of related topics with appropriate and rich citations. As such, it is thus poised to become a valuable contribution to the field.
I provide suggestions and constructive criticisms below, which are not intended to undermine my positive evaluation of this paper.

MAIN SUGGESTIONS
• The manuscript could be condensed by avoiding certain redundancies, and streamlining the parts that are very classic (e.g. Figure 1).
• Reversible vs irreversible:
o The end of section 1.1. reminds one of the distinctions between specification and commitment, which had an experimental basis in models where tissues could be grafted ectopically and/or heterochronically and either reprogram (if just specified) or maintain their identity (if committed).
o Certain irreversible transitions involving alterations of the genome could be mentioned: e.g. VDJ recombination, red blood cell differentiation in mammals,...
o Better relate reversibility with molecular underpinnings. For example a figure mapping molecular processes on a reversibility vs. "central dogma" axis (DNA-chromatin-transcription, post-transcriptional processes, ...). The paper suggests that chromatin modifications such as DNA methylation may foster more irreversible transitions, whereas certain post-transcriptional and behavioral transitions may be more reversible. Such a Figure would advantageously replace Figures 1 and/or 3, which are quite generic and/or not specifically about the topic of state transitions in development and regeneration.
• Part 3 reads more like an extension of part 2.3, about the impact of extracellular inputs on cell states, than a consideration of cell states for the tissue-scale properties/function. E.g. A multipotent progenitor state may be important in the context of morphogenetic states in vertebrate where fate decisions involve tissue-scale gradients of diffusing morphogens; likewise a cell-level migratory state underlie tissue-scale movements (this is well described in the paper).
• It would be useful to distinguish intermediate from transition "state", the latter is somewhat of an oxymoron as it refers to an unstable transition between two other stable states, a bit by analogy with chemical reactions having to go over an activation energy barrier; whereas the former may represent a stable intermediate between two other stable states, for example lineage-restricted multipotent progenitors between pluripotent stem cells and specified or committed precursors of differentiated cells.
• Potentially relevant additional concepts include, especially for intermediate states:
o Competence: Potentially important to discuss when connecting state to function. For example, the state of a premigratory cell renders it competent to migrate in response to cues. The migratory state is different from a behavioral perspective, but a migration competent state is a pre-requisite. The two may be the same from a transcriptome perspective...
o microRNAs. E.g. miR-133 inhibiting Myod translation until myogenesis proceed. The cell is primed with mRNA but no protein is produced yet. (e.g. paper by Margaret Buckingham lab)
o Paused polymerase. Rich literature about this mechanism to poise genes for (rapid) activation. (e.g. Richard Young, Mike Levine...)
o Multilineage transcriptional priming, first described in the blood lineages in 1997 and in many systems since.
o Multipotent states resolved during fate decisions by so-called regulatory cross-antagonisms. E.g. the whole GAP gene cascade in Drosophila, dorso-ventral patterning of the vertebrate neural tube. Related: transcriptional repression, potentially leading to the noted Polycomb-mediated silencing.
o Trade-offs: specialization though differentiation and maturation fosters performance. e.g. mammalian cardiomyocytes lose the potential to divide, and thus heal an injured heart, upon maturation which ensure greater contractile performance. The proliferative state of cardiomyocytes in vertebrates is directly related to their ability to regenerate cardiac tissue.

MINOR SUGGESTIONS
• On two occasions the authors seem to write "revision/revising" when they meant "review/reviewing".
• P.3: "missing tissues are repatterned" reads a bit awkward as they would need to be rebuild first.

Referee #2:

The manuscript by Saraiva and colleagues presents a discussion on cell states, which includes a more historical perspective (and definitions) followed by a discussion on the reversibility of cell state transitions and some examples of changes in chromatin accessibility that accompany those transitions. The authors also posit that similarities between developmental changes (and epigenetic changes) may be at play in regeneration models.

In general, the manuscript is easy to read, however, at instances it reads more like an opinion than a review. The authors aimed

to cover a large scope and as a result, the review presents a few chosen examples for some phenomena than an in-depth coverage of the topic. This is not necessarily bad, but it may mislead the readership and at instances, it becomes inaccurate. I would suggest that the manuscript is published as an opinion, also because throughout, the authors are not always accurate but remain very general. Below are more specific sections that need attention for accuracy and/or because they are not really backed up by data. I also have a major conceptual disagreement with the way the authors interpret chromatin accessibility versus chromatin modifications, which they sometimes use interchangeably.

Page 1 (my page numbering starts at the introduction page).
1. The historical perspective is nice !
2. (optional) Second paragraph, the authors may want to discuss the idea of a 'fluidity' of cellular states based on our ability to detect e.g. transcripts: are the (intermediate) states truly fluid ? or we just are able to detect more differences? If the latter, how relevant is this really to cell function?
3. Second paragraph, the authors mention transcriptomics, multi-omics and variations in cell populations (spatial, collective) as part of the 'cell state' make-up, but they do not include epigenetics. Considering that the 'programme' of the cell and the epigenetics is discussed at large, they should introduce epigenetics as part of the make-up of a given cellular state.
4. Second paragraph. Considering the many definitions of 'cellular states' in the community that are presented by the authors, it will be important that the authors state what THEY define as cellular state, so that the readers know what the authors refer to, throughout the manuscript.

Page 2.
1. Second paragraph. The first transition of the ontogeny occurs 'during the fusion of male and female pronuclei'. This sentence is incorrect when it is used in such a general framework: for example, in mammals, the pronuclei never fuse and it is also unclear whether this is the case in other vertebrates. Pronuclei also do not fuse in Drosophila or in C. elegans. Please correct (an alternative would be gamete fusion, which would be universal).
2. Second paragraph. The explanation of MZT-ZGA and blastula is also not applicable to mammals and as written, the statements are thus incorrect. The paragraph needs to be corrected.
3. Second paragraph. It is unclear what '3 of the transitions that occur in a developing embryo' refers to. Do the authors refer to gamete fusion, MZT and gastrulation ? if that is the case, how are placental mammals included here ? (e.g. what would be the 3 transitions ?). I fear that the paragraph aims to summarise very general processes with very specific numbers and as a result it ends up being inaccurate.

Page 3.
1. The authors write in the second paragraph that single cell technologies allowed the realisation that cell transitions are not unidirectional. I do not think this is correct, I think it was the (discovery) of reprogramming, that enabled the scientific community to conclude this: it is not the technology of single cell that discovered the reversibility of the cell states.
2. (optional) Paragraph 2, end of the last sentence. Perhaps the authors could distinguish reversibility of cell states and (un)stability of states: the states are not necessarily reversible, but rather unstable.
3. Paragraph 4. The argument that chromatin regulation is key in determining whether transitions are reversible or not is not fully founded. An alternative interpretation is that it is chromatin regulation that enables reversibility (e.g. chromatin enables, but does not determine).
4. Paragraph 4: the authors provide as an example of development, embryonic stem cells. This is misleading: while embryonic stem cells are a model to study pluripotency during development, they are not 'early development'. It is surprising that the authors use ESCs throughout as example of early development, considering the vast amount of literature on actual mouse embryos on this subject. Please either refer to development (and not to ESCs in culture) or avoid using the term 'development' in this context.

Page 5.
1. Paragraph 2. Here the authors refer again to 'genes playing instructive roles in development' by citing an example of embryonic stem cells.

Page 6.
1. Paragraph 2. The authors state that chromatin marks play instructive roles in transcriptional regulation by controlling the accessibility of TFs to their binding site. This statement is not founded and is not supported by data in the field: i) the evidence that histone modifications are instructive is poor (and in any case it is not universal); ii) to my knowledge, there is no evidence that histone modifications change chromatin accessibility, what is the evidence that the authors have to support this statement?
2. Paragraph 3. The discussion on enhancers would also need to be better phrased: while K4me1 and K27ac are enriched at potential enhancers, their presence does not mean that histone modifications 'cause' them to be enhancers (in fact many enhancers with K4me1 and K27ac are not active).

Page 7.
1. Paragraph 1. The authors refer to lineage commitment during development and refer to ESCs as an example. As stated above, ESCs are not 'development' (even though they are a proxy for development).
2. Paragraph 1. The sentence 'interestingly, this global epigenetic change does not occur in neurons until adolescence' is also misleading: the sentence reads as if there are no changes in chromatin remodelling during neuronal development, which is

incorrect. Please rephrase for accuracy.

Page 8.
1. Paragraph 2. I am not aware of literature demonstrating that folate increases methylation at brain-specific genes directly. The paper the authors cite (ref 195) presents data indicating that a folate diet results in changes in DNA methylation of some genes. The authors statement suggest that there is a direct influence of vitamins (and folate) on chromatin modifications, but I would be cautious on how this is written, since direct effects have not been demonstrated.
2. Paragraph 2. Similarly, the authors suggest that RA achieves changes in cell behaviour by inducing changes in gene expression and in epigenetic modifications, but, is it not through genetic regulation (of gene expression) that RA receptors function ? how can the authors disentangle effects on epigenetic regulation from gene expression in this case?

Page 8 and 9. I find the sections on mechanical regulation very superficial and oversimplified. For example, the LINC complex is really only one of many mediators of mechanotransduction. I would suggest the authors remove completely these parts. They are not in depth and instead they read as if just a couple of examples were 'chosen' together. I do not think the review will suffer if this part is removed.

Page 11.
1. Third paragraph. The authors state that mechanical stimuli can lead to epigenetic modifications. This is not fully reflecting the literature cited: the paper in question studies only chromatin accessibility and I would encourage the authors to more specifically/faithfully describe the findings of others in their review.

Referee #1:

SUMMARY

In this review entitled "Dynamic control of cell state transitions during embryo development and regeneration", Saraiva et al. consider the concept of cell state, especially its multimodal nature from chromatin dynamics to cellular behaviors, and the extent to which states are reversible as a function of cell-intrinsic properties and cell-extrinsic influences, whether biochemical or mechanical.

After introducing the concept of cell state being multimodal through a historical lens, connecting the concept to (cellular) function, the authors first survey classic transitions, including the maternal-to-zygotic and epithelium-to-mesenchyme transitions during development, and contrast with regeneration-specific transitions that follow a different, sometimes opposite direction from differentiated to undifferentiated states. The authors then pivot to discussing the degrees to which specific transitions are reversible, which is an intuitive premise to regenerative capabilities, providing a rich suite of examples covering various modalities of more-or-less plastic transitions, from chromatin states to cellular behaviors.

The authors then proceed to visiting the molecular underpinnings of cell state transitions, starting with gene expression followed by chromatin states and the impact of the cell microenvironment on state transitions, providing an extensive review of the relevant literature. Finally, the authors place the cell-centric concept of state into a multiscale perspective by discussing how cell state transitions contribute to tissue-scale development and morphogenesis.

The authors provide 4 Figures to illustrate their points: Figure 1 is a rather general and simple illustration of embryonic development and regeneration, Figure 2 illustrates various transitions in cell states and multicellular contexts, Figure 3 illustrates example of biochemical and mechanical signal transduction mechanisms impacting chromatin states and gene expression, Figure 4 illustrates the tissue context in which external/mechanical properties influence state transitions.

KEY MERITS AND GENERAL EVALUATION

This review addresses an active area of research, providing a very thorough, creative and inspiring perspective, covering a broad range of related topics with appropriate and rich citations. As such, it is thus poised to become a valuable contribution to the field. I provide suggestions and constructive criticisms below, which are not intended to undermine my positive evaluation of this paper.

We thank the reviewer for their inputs, their revision was extremely constructive and all points were clearly indicated with suggestions that helped the revision process. We highlight our response to their points in blue, and as there was a large number of requests, we indicate the line number in which changes were implemented.

MAIN SUGGESTIONS

• The manuscript could be condensed by avoiding certain redundancies, and streamlining the parts that are very classic (e.g. Figure 1).

We have revised the manuscript for redundancy as much as possible in both, text and figures. For example, we removed discussions about mechanical aspects of section 2.3 and moved only part of it to section 3. In this way we highlight the relevance of mechanical signalling as an example of the impact of tissue interplay in cell state transitions.

• Reversible vs irreversible:

o The end of section 1.1. reminds one of the distinctions between specification and commitment, which had an experimental basis in models where tissues could be grafted ectopically and/or heterochronically and either reprogram (if just specified) or maintain their identity (if committed).

o Certain irreversible transitions involving alterations of the genome could be mentioned: e.g. VDJ recombination, red blood cell differentiation in mammals,...
Thanks for this comment, we have added the mentioned examples and better distinguished between specification and commitment in (Lines 162-166) and the examples are provided in lines 176-181.

o Better relate reversibility with molecular underpinnings. For example a figure mapping molecular processes on a reversibility vs. "central dogma" axis (DNA-chromatin-transcription, post-transcriptional processes, ...). The paper suggests that chromatin modifications such as DNA methylation may foster more irreversible transitions, whereas certain post-transcriptional and behavioral transitions may be more reversible. Such a Figure would advantageously replace Figures 1 and/or 3, which are quite generic and/or not specifically about the topic of state transitions in development and regeneration.
We have applied this suggestion as before we were expanding in context at expenses of detail and this point truly assisted to address this issue, thanks (**see new Figure 2**). Also, please note that because the wide audience of this journal, we kept Fig 1 as generic as it can introduce new comers to biological contexts.

• Part 3 reads more like an extension of part 2.3, about the impact of extracellular inputs on cell states, than a consideration of cell states for the tissue-scale properties/function. E.g. A multipotent progenitor state may be important in the context of morphogenetic states in vertebrate where fate decisions involve tissue-scale gradients of diffusing morphogens; likewise a cell-level migratory state underlie tissue-scale movements (this is well described in the paper).
This was indeed confusing. We have now amended the structure of our article by removing the last part of 2.3 (related to mechanical signaling). These parts were then distributed along mechanistic examples of part 3 illustrating how biophysical quantities emerging from tissue interplay can also lead to a regulation of chromatin, gene expression and eventually cell states (please see new section 3). Also, examples of multipotent progenitors in morphogenesis are now added on lines 564-566.

• It would be useful to distinguish intermediate from transition "state", the latter is somewhat of an oxymoron as it refers to an unstable transition between two other stable states, a bit by analogy with chemical reactions having to go over an activation energy barrier; whereas the former may represent a stable intermediate between two other stable states, for example lineage-restricted multipotent progenitors between pluripotent stem cells and specified or committed precursors of differentiated cells.
We provide the suggested explanations on lines 185-195 of the revised manuscript.

• Potentially relevant additional concepts include, especially for intermediate states:

We added these points in the following lines:
o Competence: Potentially important to discuss when connecting state to function. For example, the state of a premigratory cell renders it competent to migrate in response to cues. The migratory state

is different from a behavioral perspective, but a migration competent state is a pre-requisite. The two may be the same from a transcriptome perspective...
Lines 196-199

o microRNAs. E.g. miR-133 inhibiting Myod translation until myogenesis proceed. The cell is primed with mRNA but no protein is produced yet. (e.g. paper by Margaret Buckingham lab)
Lines 199-202

o Paused polymerase. Rich literature about this mechanism to poise genes for (rapid) activation. (e.g. Richard Young, Mike Levine...)
Lines 202 - 205

o Multilineage transcriptional priming, first described in the blood lineages in 1997 and in many systems since
Lines 207-210

o Multipotent states resolved during fate decisions by so-called regulatory cross-antagonisms. E.g. the whole GAP gene cascade in Drosophila, dorso-ventral patterning of the vertebrate neural tube. Related: transcriptional repression, potentially leading to the noted Polycomb-mediated silencing.
Lines 210-216

o Trade-offs: specialization though differentiation and maturation fosters performance. e.g. mammalian cardiomyocytes lose the potential to divide, and thus heal an injured heart, upon maturation which ensure greater contractile performance. The proliferative state of cardiomyocytes in vertebrates is directly related to their ability to regenerate cardiac tissue –
Lines 153-159

Thanks for pointing this out as it really strengthened our points.

MINOR SUGGESTIONS

- On two occasions the authors seem to write "revision/revising" when they meant "review/reviewing".
- P.3: "missing tissues are repatterned" reads a bit awkward as they would need to be rebuild first.

We have revised the manuscript accordingly.

Referee #2:

The manuscript by Saraiva and colleagues presents a discussion on cell states, which includes a more historical perspective (and definitions) followed by a discussion on the reversibility of cell state transitions and some examples of changes in chromatin accessibility that accompany those transitions. The authors also posit that similarities between developmental changes (and epigenetic changes) may be at play in regeneration models.

In general, the manuscript is easy to read, however, at instances it reads more like an opinion than a review. The authors aimed to cover a large scope and as a result, the review presents a few chosen examples for some phenomena than an in-depth coverage of the topic. This is not necessarily bad, but it may mislead the readership and at instances, it becomes inaccurate. I would suggest that the manuscript is published as an opinion, also because throughout, the authors are not always accurate but remain very general. Below are more specific sections that need attention for accuracy and/or because they are not really backed up by data. I also have a major conceptual disagreement with the way the authors interpret chromatin accessibility versus chromatin modifications, which they sometimes use interchangeably.

We would like to thank the reviewer for their comments, they are constructive and truly assisted us to strengthen the manuscript. Their comments were not only constructive but were delivered in a clear way and with concrete examples on how to address them. Our response is in blue and we indicate the lines in which changes have been applied.

Page 1 (my page numbering starts at the introduction page).

1. The historical perspective is nice!
Thanks, we kept it in the revised version.

2. (optional) Second paragraph, the authors may want to discuss the idea of a 'fluidity' of cellular states based on our ability to detect e.g. transcripts: are the (intermediate) states truly fluid ? or we just are able to detect more differences? If the latter, how relevant is this really to cell function?
We have discussed these points in Lines 218 – 235.

3. Second paragraph, the authors mention transcriptomics, multi-omics and variations in cell populations (spatial, collective) as part of the 'cell state' make-up, but they do not include epigenetics. Considering that the 'programme' of the cell and the epigenetics is discussed at large, they should introduce epigenetics as part of the make-up of a given cellular state.
We have added this point in lines 67-69, and refer to the reader to point 2.2 where we discuss chromatin modifications and cell states.

4. Second paragraph. Considering the many definitions of 'cellular states' in the community that are presented by the authors, it will be important that the authors state what THEY define as cellular state, so that the readers know what the authors refer to, throughout the manuscript.
We have added a definition in Lines 75-76.

Page 2.

1. Second paragraph. The first transition of the ontogeny occurs 'during the fusion of male and female pronuclei'. This sentence is incorrect when it is used in such a general framework: for

example, in mammals, the pronuclei never fuse and it is also unclear whether this is the case in other vertebrates. Pronuclei also do not fuse in Drosophila or in C. elegans. Please correct (an alternative would be gamete fusion, which would be universal).
We corrected in Lines 102-103.

2. Second paragraph. The explanation of MZT-ZGA and blastula is also not applicable to mammals and as written, the statements are thus incorrect. The paragraph needs to be corrected.
We corrected in Lines 103-115.

3. Second paragraph. It is unclear what '3 of the transitions that occur in a developing embryo' refers to. Do the authors refer to gamete fusion, MZT and gastrulation ? if that is the case, how are placental mammals included here ? (e.g. what would be the 3 transitions ?). I fear that the paragraph aims to summarise very general processes with very specific numbers and as a result it ends up being inaccurate.
We corrected in Lines 103 – 126.

Page 3.

1. The authors write in the second paragraph that single cell technologies allowed the realisation that cell transitions are not unidirectional. I do not think this is correct, I think it was the (discovery) of reprogramming, that enabled the scientific community to conclude this: it is not the technology of single cell that discovered the reversibility of the cell states.
This was an important point and we amended in Lines 170-173. Indeed, the technique may not necessarily reflect a cell state as part of the protocol lead to a loss of context, so it may be grasping only part of it.

2. (optional) Paragraph 2, end of the last sentence. Perhaps the authors could distinguish reversibility of cell states and (un)stability of states: the states are not necessarily reversible, but rather unstable.
We now make this distinction in Lines 185 – 195.

3. Paragraph 4. The argument that chromatin regulation is key in determining whether transitions are reversible or not is not fully founded. An alternative interpretation is that it is chromatin regulation that enables reversibility (e.g. chromatin enables, but does not determine).
We apologise for not making this distinction, we now make it clearer in Lines 244 – 250.

4. Paragraph 4: the authors provide as an example of development, embryonic stem cells. This is misleading: while embryonic stem cells are a model to study pluripotency during development, they are not 'early development'. It is surprising that the authors use ESCs throughout as example of early development, considering the vast amount of literature on actual mouse embryos on this subject. Please either refer to development (and not to ESCs in culture) or avoid using the term 'development' in this context.
Corrected in Lines 251 – 261.

Page 5.

1. Paragraph 2. Here the authors refer again to 'genes playing instructive roles in development' by citing an example of embryonic stem cells.
Amended in Lines 334- 342.

Page 6.

1. Paragraph 2. The authors state that chromatin marks play instructive roles in transcriptional regulation by controlling the accessibility of TFs to their binding site. This statement is not founded and is not supported by data in the field: i) the evidence that histone modifications are instructive is poor (and in any case it is not universal); ii) to my knowledge, there is no evidence that histone modifications change chromatin accessibility, what is the evidence that the authors have to support this statement?
We appreciated this comment. We do see the referee's point and have toned down how we refer to the relationship of chromatin marks to repressive or permissive states rather than being an active instructive driver of transcriptional responses. We provide examples of this and refer to relevant literature. Please see Lines 375- 380; 387 – 394 of the revised manuscript.

2. Paragraph 3. The discussion on enhancers would also need to be better phrased: while K4me1 and K27ac are enriched at potential enhancers, their presence does not mean that histone modifications 'cause' them to be enhancers (in fact many enhancers with K4me1 and K27ac are not active).
This was removed to avoid confusion arising from a black and white statement about something is not. Thanks for the comment.

Page 7.

1. Paragraph 1. The authors refer to lineage commitment during development and refer to ESCs as an example. As stated above, ESCs are not 'development' (even though they are a proxy for development).
Replaced by examples from mouse embryos in Lines 419 – 422.

2. Paragraph 1. The sentence 'interestingly, this global epigenetic change does not occur in neurons until adolescence' is also misleading: the sentence reads as if there are no changes in chromatin remodelling during neuronal development, which is incorrect. Please rephrase for accuracy.
Removed to avoid confusion.

Page 8.

1. Paragraph 2. I am not aware of literature demonstrating that folate increases methylation at brain-specific genes directly. The paper the authors cite (ref 195) presents data indicating that a folate diet results in changes in DNA methylation of some genes. The authors statement suggest that there is a direct influence of vitamins (and folate) on chromatin modifications, but I would be cautious on how this is written, since direct effects have not been demonstrated.
We have rephrased this in Lines 511-516.

2. Paragraph 2. Similarly, the authors suggest that RA achieves changes in cell behaviour by inducing changes in gene expression and in epigenetic modifications, but, is it not through genetic regulation (of gene expression) that RA receptors function? how can the authors disentangle effects on epigenetic regulation from gene expression in this case?
Thanks for making this point, we amend our text and refer mostly to RA binding to RAREs and its influence on epigenetic regulation of gene expression. Please see Lines 523 – 529**.**

Page 8 and 9.

I find the sections on mechanical regulation very superficial and oversimplified. For example, the LINC complex is really only one of many mediators of mechanotransduction. I would suggest the authors remove completely these parts. They are not in depth and instead they read as if just a couple of examples were 'chosen' together. I do not think the review will suffer if this part is removed.
We now added more relevant examples in section 3 and removed those parts from section 2.3 (please see lines 567 – 576; 593-638; 686-720; 735-738). Also, we changed the figure to provide a more mechanistic overview. Please see new Figure 3 of the revised manuscript.

Page 11.

1. Third paragraph. The authors state that mechanical stimuli can lead to epigenetic modifications. This is not fully reflecting the literature cited: the paper in question studies only chromatin accessibility and I would encourage the authors to more specifically/faithfully describe the findings of others in their review.
We apologise for overseeing this, we now added more literature on the topic and we are happy to add more if needed, please see Lines 616 – 629.

Dear Elias,

Thank you for submitting the revised version of your review to The EMBO Journal. I sincerely apologise for the delay in communicating the decision due to conference travel and the resulting backlog. Your manuscript has now been seen by both original reviewers, who find that most of their comments have been sufficiently addressed.

I have now gone through the manuscript and have added a couple of minor edits or suggestions - please take a look at the attached manuscript text file and accept of modify as needed.

There are also a few additional aspects that need to be addressed in the final version:
1. In our standard text plagiarism check, we noted a couple of sentences that appear to show high similarity to a published article by other authors (attached). Please consider rephrasing these sentences (lines 700-707).
2. In figure 4B, there is a type in "Importin". Please also go through the figures and correct the cases where capital letters have been introduced (figure 3, "Nuclear & chromatin deformation"; figure 4, "Transcription & regeneration").
3. Please submit figures as separate, high-resolution image files and add figure legends to the manuscript text file.
4. Please add complete funding information in our online system, including project numbers where available.
5. Please update the headings to "Declaration of Competing Interests Statement" and "References".

Please let me know if you have any questions, and I look forward to receiving the final version of your manuscript!

With best wishes,

Ieva

Ieva Gailite, PhD
Senior Scientific Editor
The EMBO Journal
Meyerhofstrasse 1
69117 Heidelberg
i.gailite@embojournal.org

We realize that it is difficult to revise to a specific deadline. In the interest of protecting the conceptual advance provided by the work, we recommend a revision within 3 months (31st Oct 2026). Please discuss the revision progress ahead of this time with the editor if you require more time to complete the revisions.

Referee #1:

in this revised version, the authors took feedbacks to heart and improved an already compelling review, producing a version that should serve as an excellent reference for many researchers in this dynamic field.
minor details might need fixing, such as some duplicated citations, but the proof reading steps should suffice.

Referee #2:

The authors have addressed the factual errors that I pointed in my original review.

The authors addressed the remaining editorial issues.

2nd Revision - Editorial Decision 19th Aug 2026

Dear Elias,

Thank you for addressing the final editorial and formatting requests. I am now pleased to inform you that your manuscript has been accepted for publication in the EMBO Journal. Congratulations on an excellent article!

Your manuscript will be processed for publication by EMBO Press. It will be copy edited and you will receive page proofs prior to publication. Please note that you will be contacted by Springer Nature Author Services to complete licensing information.

Should you be planning a Press Release on your article, please notify the production team via this form in order to coordinate publication and release dates: https://forms.cloud.microsoft/e/eH4A8JVP6g

If you have any questions, please do not hesitate to contact the Editorial Office. Thank you for this thought-provoking contribution to The EMBO Journal!

With best wishes,

Ieva

Ieva Gailite, PhD
Senior Scientific Editor
The EMBO Journal
Meyerhofstrasse 1
69117 Heidelberg
i.gailite@embojournal.org

*****
Please note that it is The EMBO Journal policy for the transcript of the editorial process (containing referee reports and your response letters) to be published as an online supplement to each paper. If you should prefer removal of any referee-only figures included in the point-by-point response(s), e.g. because they may still be used for future publication or because they have been reproduced from published work by others, please do let us know immediately via response email.
More information is available here: https://link.springer.com/partners/embo-press/editorial-policies#Peer%20review
